# Mutated *DNMT3A* creates a public HLADQ- binding neoantigen on acute myeloid leukemia

**DOI:** 10.3389/fimmu.2025.1556121

**Published:** 2025-03-13

**Authors:** Dyantha I. van der Lee, Eva M. Argiro, Sebastiaan N. J. Laan, M. Willy Honders, Rob C. M. de Jong, Nadine E. Struckman, J. H. Frederik Falkenburg, Marieke Griffioen

**Affiliations:** Department of Hematology, Leiden University Medical Center, Leiden, Netherlands

**Keywords:** acute myeloid leukemia, immunotherapy, cancer immunity, neoantigen, HLA class II, CD4 T cell, DNMT3A, *RUNX1*

## Abstract

**Introduction:**

Patients with acute myeloid leukemia (AML) often carry the same gene mutations. Neoantigens encoded by these mutations are attractive targets for immunotherapy.

**Methods:**

We searched for public human leukocyte antigen (HLA) class II-restricted neoantigens on AML using an *in vitro* T cell stimulation method. Peptides from 26 recurrent genetic aberrations were assessed for predicted HLA class II binding, and 24 long neopeptides encoded by 10 recurrent mutations were synthesized. Naive CD4 T cells from healthy individuals were cocultured with autologous dendritic cells pulsed with neopeptides.

**Results:**

Multiple CD4 T cell clones were isolated that recognized neopeptides encoded by 5 different genetic aberrations. Two of these peptides, one from the well-known *DNMT3A-R882H* hotspot mutation and one from a long alternative reading frame created by frameshift mutations in *RUNX1*, were recognized by CD4 T cell clones after endogenous processing and presentation on cell lines transduced or CRISPR-Cas9-edited with the mutation of interest. The T cell clone for DNMT3A-R882H was also activated upon stimulation with primary AML samples from HLA-DQB1*06:02 or -DQB1*06:03 positive patients with the mutation.

**Conclusion:**

We here identified a public HLA class II-restricted neoantigen encoded by a driver mutation occurring in 10% of patients with AML that could become an important target for immunotherapy to treat patients with *DNMT3A-R882H*-mutated AML.

## Introduction

Acute myeloid leukemia (AML) is a malignancy of the myeloid lineage caused by disrupted maturation and uncontrolled expansion of hematopoietic progenitor cells. Current treatment consists of (combinations of) chemotherapy, hypomethylating agents or targeted small molecule inhibitors, which can be followed by allogeneic hematopoietic stem cell transplantation in patients with high risk AML (1, 2). Although most patients initially enter complete remission, relapses occur in approximately half of the patients (3). To improve overall survival, novel therapies are needed to effectively treat AML.

T cell-based immunotherapies targeting different tumor antigens are actively being explored for the treatment of AML (4). Selecting the right target antigen is crucial to ensure strong anti-tumor effects while minimizing side effects (5). Identifying suitable target antigens on AML, however, remains a major challenge. So far, clinical trials have focused on lineage antigens, which are restricted to myeloid cells, and leukemia-associated antigens, which are overexpressed on AML (4, 6). While these targets often allow treatment of relatively large patient populations, they also carry a significant risk for on-target off-tumor toxicity, where antigen-expressing healthy cells are also attacked (6). For example, patients with AML treated with chimeric antigen receptor-engineered T cells against myeloid lineage antigens such as CD33, CD123 or CLEC12A have experienced myelosuppression due to damage to healthy hematopoietic cells (7). To overcome these challenges, researchers are actively searching for novel antigens that are more AML-specific to enhance therapeutic precision and safety.

Neoantigens are peptides presented on tumor cells by human leukocyte antigens (HLA) that are recognized by T cells. They arise from tumor-specific somatic mutations and are expressed only on malignant cells, making them attractive targets for immunotherapy. However, only a small fraction of tumor-specific mutations generate neoantigens, and these antigens are therefore more common on tumors with a high mutational burden, such as melanoma and lung carcinoma (8). Although AML has a relatively low number of mutations, with an average of 10–13 coding mutations per patient (9, 10), many mutations are recurrent and shared across different patient populations (9, 11). Moreover, many recurrent genetic aberrations in AML are driver mutations that play a key role in tumor development and progression (12). As a result, targeting neoantigens encoded by recurrent mutations presents a promising new approach for immunotherapy of AML.

Various groups have identified HLA class I-restricted neoantigens on AML (13–22). We (15, 16) and others (18–20) have shown that specific T cell receptors (TCRs) for HLA class I neoantigens can be used to target AML *in vitro* and in mice. CD8 T cells release cytotoxic molecules upon recognizing antigens presented by HLA class I molecules, which are expressed on most tumor cells, making them optimal tools for targeting tumors. However, CD4 T cells play an essential and more indirect role in anti-cancer immunity by promoting CD8 T cell priming, stimulating their effector and memory functions and overcoming immunosuppression (23, 24). Some CD4 T cells are also capable of directly killing HLA class II-expressing tumor cells (25). Since AML cells often express HLA class II on their surface, it is worth exploring the potential of CD4 T cells to target HLA class II-restricted neoantigens on AML.

In this study, we aimed to identify HLA class II-restricted neoantigens encoded by recurrent driver mutations in AML. We analyzed 26 recurrent genetic aberrations for encoding peptides with predicted HLA class II binding and synthesized 24 long neopeptides created by 9 recurrent driver mutations in *DNMT3A*, *FLT3*, *IDH1*, *IDH2*, *KIT*, *NRAS*, *RUNX1* and *NPM1* and one recurrent *NUP98::NSD1* gene fusion. Naive CD4 T cells from healthy individuals were stimulated with dendritic cells pulsed with mixes of long neopeptides, and various neopeptide-specific CD4 T cell clones were isolated. Two neopeptides, one encoded by the *DNMT3A-R882H* hotspot mutation and one derived from a long alternative reading frame created by *RUNX1* frameshift mutations, were shown to be endogenously processed and presented on the cell surface. One CD4 T cell clone also reacted against multiple patient-derived *DNMT3A*-mutated AML, thereby confirming that this mutation encodes an HLA class II neoantigen on AML.

## Materials and methods

### Peptide selection

A total of 26 recurrent genetic aberrations in AML were selected based on mutation frequencies reported in the COSMIC database (26). The genetic aberrations were searched for encoding peptides with predicted HLA class II binding using the algorithm NetMHCIIpan 3.2 (27). Peptide sequences of 12-17 amino acids were entered into the algorithm to predict binding to 23 common HLA class II beta alleles with respective alpha alleles as available in the algorithm. The 23 HLA class II beta alleles are each present at an allele frequency of 10% in at least one population from different world regions as specified in the Allele Frequency Net Database (28). Strong and weak binding peptides as defined by default thresholds of ≤2% and ≤10% rank, respectively, were selected, and partially overlapping length variants were combined *in silico* into 68 unique long sequences. Of these 68 sequences, 16 long peptides comprising sequences with predicted binding to at least 4 HLA class II beta alleles were synthesized and used for T cell stimulation.

### Cell culture

Bone marrow and peripheral blood mononuclear cells (PBMCs) were collected from patients with AML and healthy individuals using density gradient centrifugation with Ficoll-amidotrizoate (pharmacy Leiden University Medical Center) and cryopreserved. Patient-derived AML samples were cultured in Iscove’s Modified Dulbecco’s Medium (IMDM) (Lonza) with 10% heat-inactivated human serum (HS) (Sanquin Reagents) and 1% penicillin/streptomycin (Lonza). B cells and monocytes were isolated from PBMCs by magnetic-activated cell sorting (MACS) using CD19 MicroBeads and CliniMACS CD14 Reagent (Miltenyi Biotec), respectively. Epstein-Barr virus-transformed lymphoblastoid cell lines (EBV-LCL) were generated from B cells. EBV-LCL and myeloid leukemia cell line K562 (ATCC), which lacks HLA class I and class II surface expression, were cultured in IMDM with 10% heat-inactivated fetal bovine serum (FBS) (Gibco), 1.5% L-glutamine (Lonza) and 1% penicillin/streptomycin. The monocytic leukemia cell line SIG-M5 (DSMZ) was cultured in IMDM with 20% FBS and 1% penicillin/streptomycin. All cell lines were regularly tested for mycoplasma using the MycoAlert Mycoplasma Detection Kit (Lonza).

### CRISPR-Cas9 genome editing of cell lines

Frameshift mutations were introduced in *RUNX1* (exon 7 in ENST00000300305.7) in K562 and SIG-M5 by CRISPR-Cas9 genome editing to generate the same alternative reading frame as created by the *c.883dup* mutation. Two CRISPR RNA sequences (crRNA) (iD1: GGCGUUGCUGGGUGCACAGA, iD4: CUGGCACGUCCAGGUGAAAU) were selected using the online tool inDelphi (29), and on- and off-targeting potential of crRNAs was assessed with the CRISPR-Cas9 guide RNA design checker tool from Integrated DNA Technologies (IDT, https://www.idtdna.com/SciTools). Ribonucleoprotein complexes (RNPs) were produced by combining crRNA with trans-activating CRISPR RNA (tracrRNA) and S*treptococcus pyogenes* Cas9 nuclease (all ordered from IDT) as previously described with modifications (30, 31). In short, crRNA and tracrRNA were mixed at a 1:1 ratio, heated for 5 minutes at 95°C and cooled to room temperature. Cas9 nuclease was added at a 1:3 ratio (30 pmol Cas9 to 90 pmol crRNA:tracrRNA duplex per transfection) and incubated for 10-20 minutes at room temperature to form RNPs. Transfection of cells was performed with the Neon Transfection System (Invitrogen). Cas9 Electroporation Enhancer (IDT) was added to RNPs to 11 µM. Per transfection, 1.1x10^6^ cells were resuspended in 110 µl Resuspension Buffer R and added to 4 µl RNPs. Using Neon Tips, 10 or 100 µl of cells and RNPs were transferred to Electroporation Tubes containing 3 ml Electrolytic Buffer E2. K562 was transfected using 1450 V, 10 ms, 3 pulses and SIG-M5 using 1200 V, 10 ms, 4 pulses. Electroporated cells were single cell cultured and clones were screened for introduced mutations by PCR and Sanger sequencing (Leiden Genome Technology Center, Leiden University Medical Center). Two clones were selected based on their introduced mutations, i.e. K562-E4 containing a homozygous *c.907dup* mutation (p.Ser303Phefs*297, iD1) and SIG-M5-C9 containing a heterozygous *c.906_907insG* mutation (p.Ser303Valfs*297, iD4). Expression of *RUNX1* mutations was confirmed by RNA sequencing as previously described (32).

### Retroviral transduction of cell lines

Cell lines K562, K562-E4 and SIG-M5-C9 were retrovirally transduced with two LZRS or MP71.60 constructs, one encoding HLA-DRB1*03:01, -DRB1*04:01, -DRB1*04:04 or -DRB1*15:01 and one encoding HLA-DRA*01:02, one encoding HLA-DQB1*03:02 and one encoding HLA-DQA1*03:01, or single constructs encoding HLA-DQA1*02:01 and -DQB1*03:03, HLA-DQA1*01:02 and -DQB1*06:02, or HLA-DQA1*01:03 and -DQB1*06:03 in which the alpha and beta chains were linked by a T2A protein cleavage site. Cell lines were also transduced with LZRS-IRES-NGFR constructs containing full-length mutated *FLT3* (p.Asp835Tyr), wild-type *FLT3*, mutated *DNMT3A* (p.Arg882His), wild-type *DNMT3A*, mutated *RUNX1* (p.Ser295Phefs*305), wild-type *RUNX1* or the full-length fusion gene *NUP98::NSD1*. Transduced cells were purified by MACS or fluorescence-activated cell sorting (FACS) using PE- (catalog 557196, BD Biosciences) or APC-conjugated NGFR (catalog CL10013APC, Sanbio) and PE-conjugated HLA-DR (catalog 347400, BD Biosciences) or HLA-DQ antibodies (catalog 318106, Biolegend).

### Neopeptide-specific CD4 T cell isolation

Naive CD4 T cells were isolated by MACS from PBMCs of healthy female individuals using the Naive CD4+ T Cell Isolation Kit II (Miltenyi Biotec). By selecting naive CD4 T cells, regulatory CD4 T cells, which mainly reside in the memory T cell pool, were largely depleted (33). Moreover, naive T cells have not yet encountered their specific antigen and represent a broad repertoire of potential antigen specificities (34). Autologous mature dendritic cells were generated by culturing monocytes for 5 days in medium with GM-CSF (Novartis Pharma) and IL-4 (Schering-Plough), followed by two days of culture in medium with GM-CSF, TNF-α (CellGenix), IL-1β (Sclavo), IL-6 (Sandoz), PGE_2_ (Sigma-Aldrich) and IFN-γ (Boehringer Ingelheim), as previously described (35). Autologous mature dendritic cells were pulsed for 4 hours with peptide pools containing each peptide at 100 nM in IMDM with 2% FBS at a concentration of 10x10^6^ dendritic cells/ml. Dendritic cells were washed and cocultured with naive CD4 T cells at a T cell:dendritic cell ratio of 10:1 in IMDM with 5% HS, 5% FBS, 2% L-glutamine, 1% penicillin/streptomycin, 10 ng/ml IL-12 (Hoffmann-La Roche) and 20 IU/ml IL-2 (Novartis Pharma) at a concentration of 1x10^6^ T cells/ml. On days 3 and 6, 100 IU/ml IL-2 and 10 ng/ml IL-15 (Miltenyi Biotec) were added. After two weeks, autologous PBMCs pulsed with the same peptide pools were irradiated at 40 Gy and added to CD4 T cells at a T cell:PBMC ratio of 2:1 in IMDM with 5% HS, 5% FBS and 10 IU/ml IL-2 at a concentration of 1x10^6^ T cells/ml. Two days after restimulation with peptide-pulsed PBMCs, activated CD137 positive CD4 T cells were bulk sorted by FACS using CD4-Pacific Blue (catalog 558116) and CD137-APC (catalog 550890) antibodies. PE-conjugated antibodies against CD8 (catalog 555367), CD14 (catalog 345785), CD16 (catalog 555407) and CD19 (catalog 345777) (all from BD Biosciences) were used for exclusion. Sorting was performed with a BD FACS Aria II 3L or BD FACS Aria III 4L cell sorter using BD FACSDiva software (BD Biosciences) at the Flow cytometry Core Facility of the Leiden University Medical Center. Sorted T cells were seeded at three cells per well in 384 well flat bottom tissue culture plates (Greiner Bio-One) containing 30 µl T cell medium consisting of IMDM with 5% HS, 5% FBS, 1.5% L-glutamine, 1% penicillin/streptomycin and 100 IU/ml IL-2, with 50,000 irradiated (40 Gy) allogeneic PBMCs as feeder cells and 0.8 µg/ml phytohemagglutinin (PHA, Thermo Scientific). Growing T cell clones were restimulated every 14-21 days with irradiated allogeneic PBMCs at a T cell:feeder cell ratio of 1:5 in T cell medium with 0.8 µg/ml PHA.

### TCR Vbeta analysis

TCR Vbeta usage of RUNX1-specific T cell clones was determined by flow cytometry using the Beta Mark TCR Vbeta Repertoire Kit (catalog IM3497, Beckman Coulter).

### T cell recognition assays

Assays were performed in 384 well flat or 96 well round bottom (Corning Life Sciences) tissue culture plates. In 384 well plates, 2,000 T cells were incubated with 10,000 stimulator cells in 50-60 µl T cell medium. In 96 well plates, T cells were incubated with stimulator cells in 100 µl T cell medium at two different ratios. For assays with HLA- and gene-transduced K562, 5,000 T cells were incubated with 60,000 stimulator cells. For assays with K562-E4, SIG-M5-C9 and patient-derived AML cells, 50,000 T cells were incubated with 100,000 stimulator cells. Release of IFN-γ or GM-CSF in supernatant was measured after overnight coincubation by enzyme-linked immunosorbent assay (ELISA) (Invitrogen, R&D Systems or Diaclone SAS). Peptide loading of stimulator cells was performed for 4 hours at 37°C in IMDM with 2% FBS. One day before use in assays, AML samples were thawed and dead cells were removed using Ficoll-amidotrizoate. Enrichment of HLA class II-expressing AML cells was performed with MACS using PE-conjugated HLA-DP antibody (catalog H130, Leinco Technologies) and anti-PE MicroBeads (Miltenyi Biotec). HLA-DP was used to avoid possible interference of anti-HLA-DQ antibodies in the interaction between the TCR and peptide-HLA-DQ complex.

### Flow cytometry-based cytotoxicity assay

For cytotoxicity assays, 50,000 T cells were incubated with 50,000 AML cells in 100 µl of IMDM without phenol red (Gibco) with 10% HS, 1.5% L-glutamine, 1% penicillin/streptomycin and 100 IU/ml IL-2 in 96 well round bottom tissue culture plates. AML cells were thawed on the day of the assay and dead cells were removed using Ficoll-amidotrizoate. Peptide loading of AML cells was performed for 2 hours at 37°C in IMDM with 2% FBS. After 48 hours of coincubation, samples were stained for 15 minutes at room temperature with viability dye Zombie Red (catalog 423109, Biolegend) and blocked for 15 minutes at room temperature with PBS (Fresenius Kabi) with 2.5% HS. Following blocking, samples were stained for 30 minutes at 4°C with CD33-PE (catalog 303404, Biolegend), CD34-APC (catalog 343510, Biolegend), HLA-DQ-FITC (catalog 318104, Biolegend), CD3-BV421 (catalog 562427, BD Biosciences), CD8-BV421 (catalog 344747, Biolegend) and CD19-BV421 (catalog 302233, Biolegend) antibodies. Samples were fixed with 1% paraformaldehyde (pharmacy Leiden University Medical Center) for 20 minutes at room temperature before acquisition on the Aurora 5L-2 (Cytek Biosciences). Results were analyzed using OMIQ (Dotmatics).

### Statistics

Statistical analyses as indicated in figure legends were performed using GraphPad Prism Software version 10.2.3 (GraphPad, Dotmatics). P-values < 0.05 were considered significant (p < 0.05 *; p < 0.01 **; p < 0.001 ***).

### Study approval

This study was approved by the Institutional Review Board of the Leiden University Medical Center (approval number B16.039). Peripheral blood and bone marrow samples were collected from patients after informed consent according to the Declaration of Helsinki and stored in the Leiden University Medical Center Biobank for Hematological Diseases.

## Results

### Selection of peptides encoded by recurrent mutations in AML

**Figure 1:**
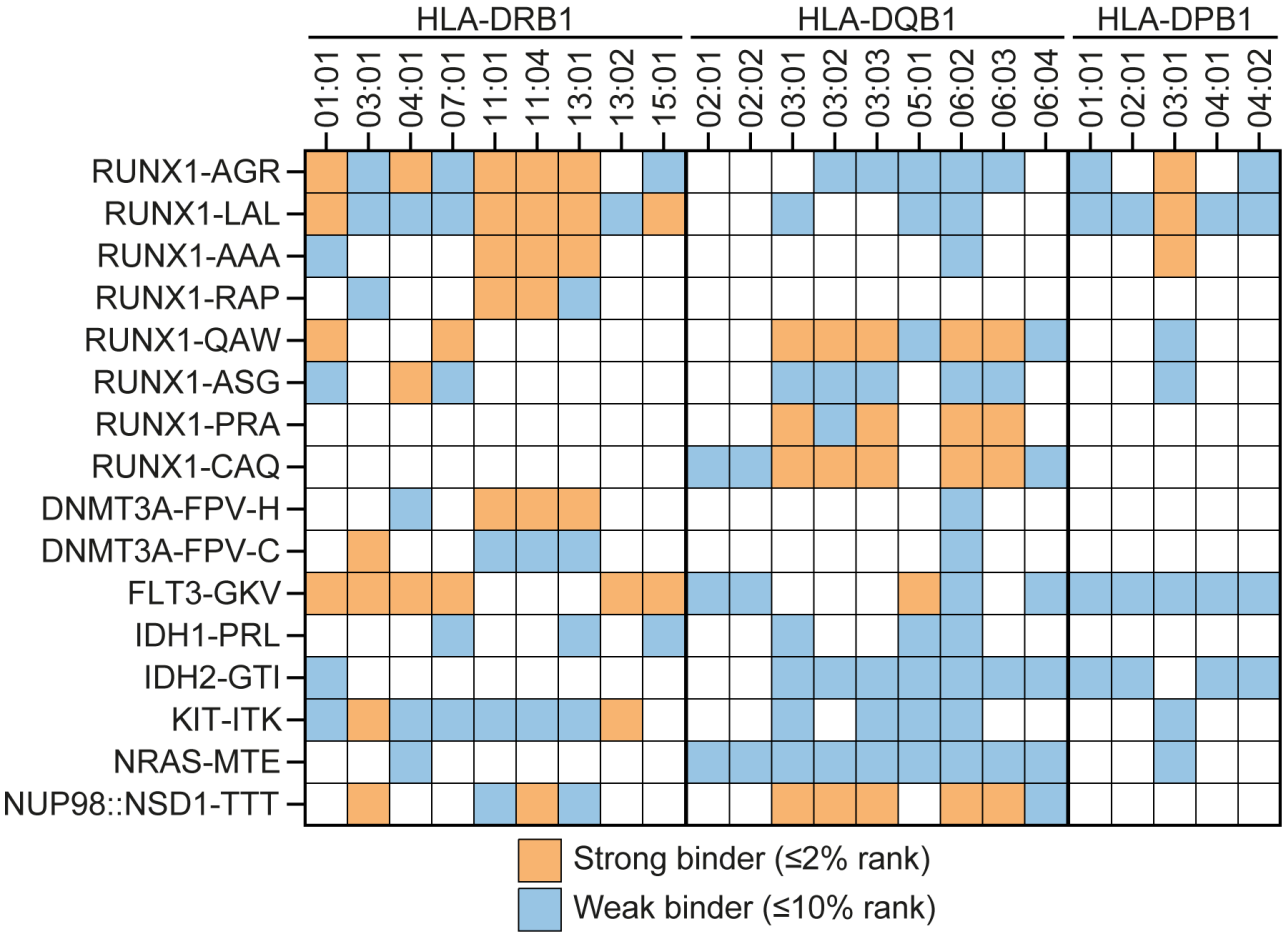
Selection of 16 neopeptides with predicted HLA class II binding. Sequences of 12-17 amino acids encoded by recurrent mutations in AML were analyzed by NetMHCIIpan 3.2 to predict binding affinity to 23 common HLA-DRB1, -DQB1 or -DPB1 alleles paired with respective HLA-DRA, -DQA1 or -DPA1 alleles as available in the algorithm. Peptides with strong or weak predicted binding were identified using default thresholds of ≤2% and ≤10% rank, respectively. A total of 16 neopeptides (18- to 28-mer peptides), annotated by their encoding gene and first three N-terminal amino acids, were synthesized. Depicted is strong (orange squares) or weak (blue squares) predicted binding to 23 HLA-DRB1 (n=9), -DQB1 (n=9) and -DPB1 (n=5) alleles in combination with at least one respective alpha allele. White squares indicate that predicted binding was >10% rank for all alpha-beta allele combinations.

A total of 26 recurrent genetic aberrations in AML were searched for encoding peptides with predicted binding to 23 common HLA class II alleles using the online tool NetMHCIIpan 3.2 (27). These aberrations included 12 missense mutations in *DNMT3A*, *FLT3*, *IDH1*, *IDH2*, *KIT* and *NRAS*, 5 small insertions or deletions leading to frameshifts in *ASXL1*, *CEBPA*, *NPM1* and *RUNX1* and 9 gene fusions involving the genes *CBFB*, *MYH11*, *KMT2A*, *MLLT3*, *NUP98*, *NSD1*, *PML*, *RARA*, *RUNX1* and *RUNX1T1* (**Supplementary Tables 1**, **2**). Based on these results, 16 long peptides each comprising sequences with weak or strong predicted binding to at least 4 HLA class II beta alleles were selected and synthesized (**Table 1**, **Figure 1**). These neopeptides are designated by their encoding gene followed by the first three N-terminal amino acids. Of these 16 neopeptides, 7 peptides were encoded by the missense mutations *DNMT3A-R882H*, *DNMT3A-R882C*, *FLT3-D835Y*, *IDH1-R132H*, *IDH2-R140Q*, *KIT-D816V* and *NRAS-G12D*, 8 peptides were derived from a long alternative reading frame generated by frameshift mutations in *RUNX1* and one peptide originated from a gene fusion between exon 12 of *NUP98* and exon 7 of *NSD1*. The median number of HLA class II beta alleles to which the neopeptides were predicted to bind was 9.5 (range 4 – 17), and strong predicted binding to at least one beta allele was observed for 13 of the 16 peptides. Of the 23 common HLA class II beta alleles, all alleles were predicted to bind to at least three neopeptides, and HLA-DQB1*06:02 was predicted to bind to a maximum of 15 neopeptides. Weak binding to at least one neopeptide was predicted for all 23 beta alleles and strong binding to at least one neopeptide for 16 of the 23 alleles. In addition to the 16 neopeptides, 8 overlapping 17-mer peptides spanning the entire alternative reading frame of 11 amino acids created by *NPM1* mutations were included irrespective of predicted HLA class II binding. *NPM1* mutations are 4 base pair insertions occurring in the final exon of the gene in 30-35% of patients with AML. As controls, peptides were synthesized for two H-Y antigens, i.e. LB-ZFY-1 and LB-RPS4Y-1 both binding to HLA-DRB1*03:01, and three minor histocompatibility antigens as well as their allelic variants, i.e. LB-LY75-2R/LB-LY75-2K, LB-MTHFD1-1Q/LB-MTHFD1-1R and LB-PTK2B-1T/LB-PTK2B-1K binding to HLA-DPB1*04:01, -DRB1*03:01 and -DRB3*01:01, respectively. The H-Y and minor histocompatibility antigens have been identified as T cell targets in patients with hematological malignancies after allogeneic hematopoietic stem cell transplantation and have thus been confirmed to be immunogenic *in vivo* (35, 36). In conclusion, 32 peptides, including 8 control antigens, were selected to stimulate CD4 T cells (**Table 1**).

**Table 1:** Peptides selected for CD4 T cell stimulation.

Peptide sequence^1^	Gene	Origin	Position	Length (AA)
**AGRPVPSQLALLPPVLRRLGRLLPV**	*RUNX1*	Frameshift	*c.883dup*	25
**LALLPPVLRRLGRLLPVLHGGRRALAAA**	*RUNX1*	Frameshift	*c.883dup*	28
**AAAHPAALHQRLHRLRAAQPQPPE**	*RUNX1*	Frameshift	*c.883dup*	24
**RAPGGGRVEALLRRQAWPGWAP**	*RUNX1*	Frameshift	*c.883dup*	22
**QAWPGWAPRAAAFASGRAGLLFATSPP**	*RUNX1*	Frameshift	*c.883dup*	27
**ASGRAGLLFATSPPGSRALGPATVLG**	*RUNX1*	Frameshift	*c.883dup*	26
**PRAPDGQDLAVGQARAASCAQKPTPP**	*RUNX1*	Frameshift	*c.883dup*	26
**CAQKPTPPPSAGAPALAEVSEATHLEGV**	*RUNX1*	Frameshift	*c.883dup*	28
FPVHYTDVSNMS**H**LARQRLLGRSWSVP	*DNMT3A*	Missense	*c.2645G>A*	27
FPVHYTDVSNMS**C**LARQRLLGRSWSVP	*DNMT3A*	Missense	*c.2644C>T*	27
GKVVKICDFGLAR**Y**IMSDSNYVVRGNAR	*FLT3*	Missense	*c.2503G>T*	28
PRLVSGWVKPIIIG**H**HAYG	*IDH1*	Missense	*c.395G>A*	19
GTI**Q**NILGGTVFREPIIC	*IDH2*	Missense	*c.419G>A*	18
ITKICDFGLAR**V**IKNDSNYVVKGNAR	*KIT*	Missense	*c.2447A>T*	26
MTEYKLVVVGA**D**GVGKSALTIQLI	*NRAS*	Missense	*c.35G>A*	24
TTTATLGFGAPQAPV**A**VRSEKKRLRKPS	*NUP98::NSD1*	Fusion gene	*NUP98 exon 12-NSD1 exon 7*	28
CFRMTDQEAIQDL**CLAV**	*NPM1*	Frameshift	*c.860_863dup*	17
FRMTDQEAIQDL**CLAVE**	*NPM1*	Frameshift	*c.860_863dup*	17
RMTDQEAIQDL**CLAVEE**	*NPM1*	Frameshift	*c.860_863dup*	17
MTDQEAIQDL**CLAVEEV**	*NPM1*	Frameshift	*c.860_863dup*	17
TDQEAIQDL**CLAVEEVS**	*NPM1*	Frameshift	*c.860_863dup*	17
DQEAIQDL**CLAVEEVSL**	*NPM1*	Frameshift	*c.860_863dup*	17
QEAIQDL**CLAVEEVSLR**	*NPM1*	Frameshift	*c.860_863dup*	17
EAIQDL**CLAVEEVSLRK**	*NPM1*	Frameshift	*c.860_863dup*	17
**QIVVEIQEAVFVSNIVDSDI**	*ZFY*	Male-specific gene	n.a.	20
**EKTGEHFRLVYDTKGRFAVH** (36)	*RPS4Y*	Male-specific gene	n.a.	20
RPTIKNE**R**FLAGLS (36)	*LY75*	SNP	*c.4040A>G*	14
RPTIKNE**K**FLAGLS (36)	*LY75*	SNP	*c.4040G>A*	14
HGNSSIIAD**Q**IALKLV (35)	*MTHFD1*	SNP	*c.1958G>A*	16
HGNSSIIAD**R**IALKLV (35)	*MTHFD1*	SNP	*c.1958A>G*	16
PMVYMND**T**SPLTPEK (35)	*PTK2B*	SNP	*c.2513A>C*	15
PMVYMND**K**SPLTPEK (35)	*PTK2B*	SNP	*c.2513C>A*	15

n.a., not applicable.

^1^Bold type amino acids (AA) represent residues translated in alternative reading frames in neopeptides created by frameshift mutations in *RUNX1* and *NPM1*, a residue indicating the position of fusion between exon 12 of *NUP98* and exon 7 of *NSD1* with both fusion partners translated in the normal reading frame, mutated residues in neopeptides encoded by missense mutations in *DNMT3A*, *FLT3*, *IDH1*, *IDH2*, *KIT* and *NRAS*, H-Y antigens LB-ZFY-1 and LB-RPS4Y-1 and polymorphic residues in minor histocompatibility antigens (LB-LY75-2R, LB-MTHFD1-1Q, LB-PTK2B-1T) and their allelic variants encoded by single nucleotide polymorphisms (SNPs).

### Isolation of neopeptide-specific CD4 T cell clones

**Figure 2:**
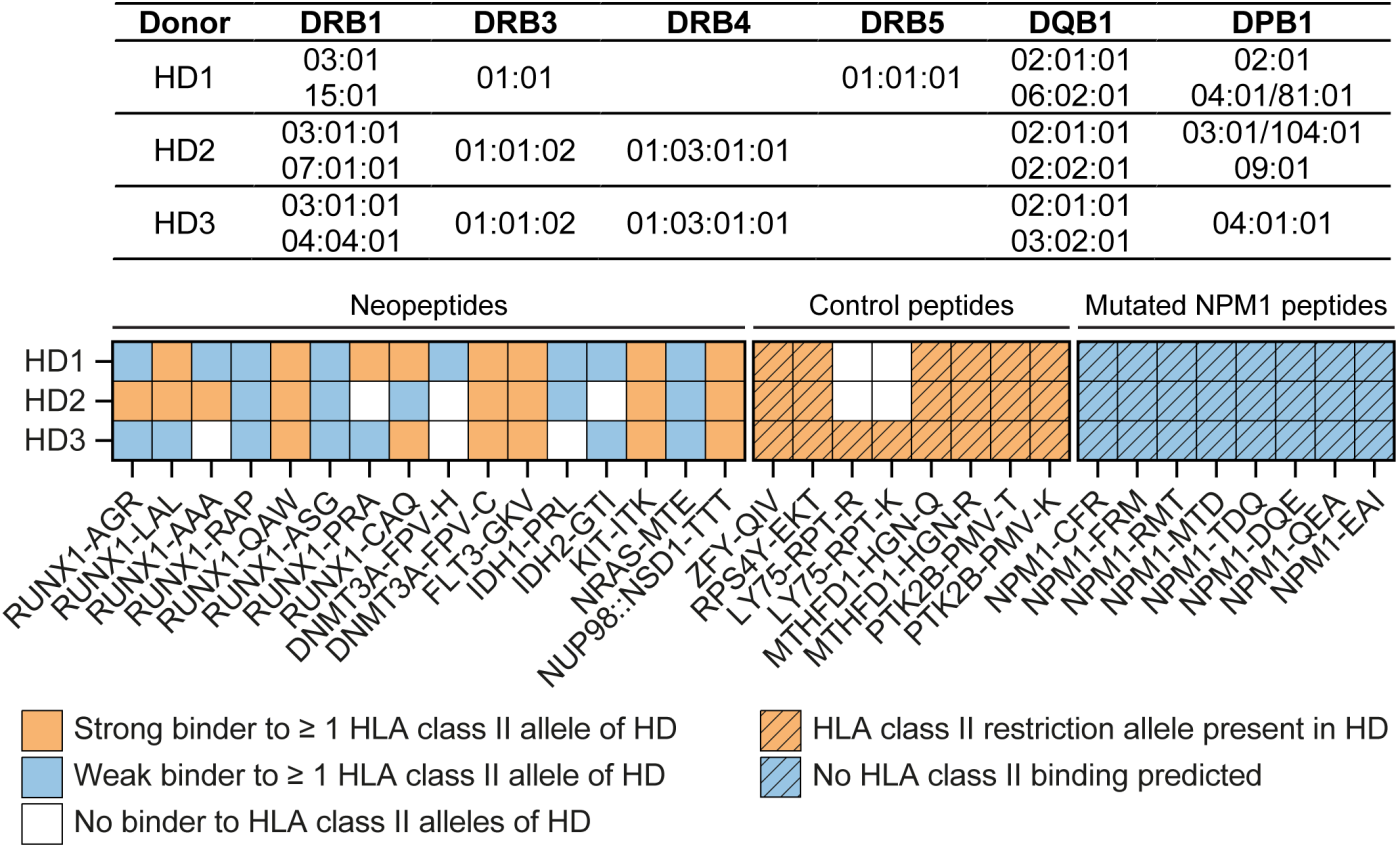
Peptide mixes used for T cell stimulation. CD4 T cells from healthy female individuals (HD1-3) were incubated with autologous dendritic cells pulsed with mixes of 27-30 peptides. Peptide mixes included 13-16 neopeptides with predicted HLA class II binding, 8 overlapping peptides spanning the entire 11 amino acid alternative reading frame of mutated NPM1 and 6-8 peptides for previously identified HLA class II-binding polymorphic antigens, including H-Y antigens, minor histocompatibility antigens and allelic variants. Donors HD1, HD2 and HD3 expressed 6, 5 and 4 of the 23 common HLA class II beta alleles, respectively, with all three donors expressing HLA-DRB1*03:01 and HLA-DQB1*02:01. Neopeptides were mixed according to their predicted binding to the HLA class II alleles of the donors. Peptides with strong (orange squares) or weak (blue squares) predicted binding to at least one HLA class II allele are indicated for each donor, and peptides that were not predicted to bind to any HLA class II allele of the donor were excluded from the mix (white squares). All 8 control peptides (patterned orange squares) were included in mixes for each of the three donors, except for peptide LY75-RPT-R and its allelic variant LY75-RPT-K, which were only used for HD3. NPM1 peptides (patterned blue squares) were included in each peptide mix irrespective of predicted HLA class II binding.

CD4 T cells from three healthy female individuals (donors HD1-3) were incubated with autologous mature dendritic cells exogenously pulsed with 27-30 peptides mixed based on predicted binding to the HLA class II alleles of the donors (**Figure 2**). Donors HD1, HD2 and HD3 expressed 6, 5 and 4 of the 23 common HLA class II beta alleles, respectively. All three donors were positive for HLA-DRB1*03:01 and HLA-DQB1*02:01. Strong binding to at least one HLA class II allele of donor HD1 was predicted for 8 neopeptides and weak binding was predicted for 8 other neopeptides in the mix. For donor HD2, 13 neopeptides were included in the mix, including 8 peptides with strong and 5 peptides with weak predicted HLA class II binding. The peptide mix for donor HD3 contained 13 neopeptides, including 6 peptides with strong and 7 peptides with weak predicted HLA class II binding. Additionally, each peptide mix contained all 8 mutated NPM1 peptides irrespective of predicted HLA class II binding and 6-8 control peptides for polymorphic antigens (H-Y antigens, minor histocompatibility antigens and allelic variants). After two weeks of culture, activated CD137 positive CD4 T cells were sorted by flow cytometry and expanded as single T cell clones. From each donor, 17,280 single T cells were cultured, and 540-936 T cell clones were isolated. Growing T cell clones were tested for reactivity against autologous EBV-LCL pulsed with peptides divided in three mixes containing mutated NPM1 peptides, other neopeptides or polymorphic peptides (**Supplementary Figure 1**). A total of 16 clones specifically reacted against peptide-pulsed EBV-LCL. Of these T cell clones, 5 clones from donor HD1 and 8 clones from donor HD3 recognized the mix of neopeptides, and one clone from donor HD2 and two clones from donor HD3 reacted against the mix of polymorphic peptides. None of the T cell clones were reactive against EBV-LCL pulsed with mutated NPM1 peptides.

**Figure 3:**
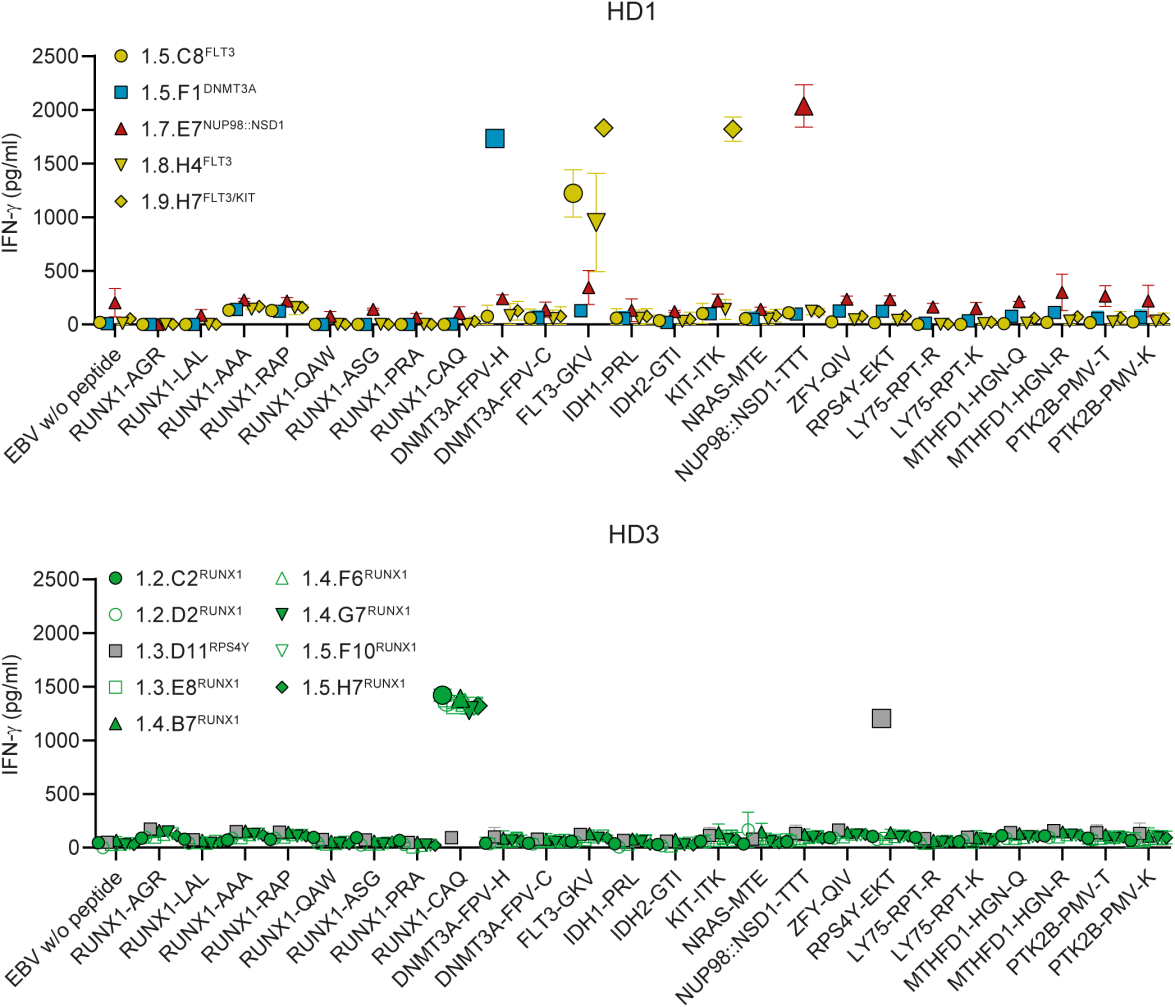
CD4 T cell clones recognize neopeptides encoded by recurrent mutations in AML. CD4 T cells from healthy donors HD1-3 were stimulated with autologous dendritic cells pulsed with mixes of neopeptides and polymorphic peptides as shown in **Figure 2**. After two weeks of culture, activated CD137 positive CD4 T cells were sorted, and single T cell clones were tested for recognition of peptide pools containing neopeptides, mutated NPM1 peptides or polymorphic peptides as depicted in **Supplementary Figure 1**. The 16 peptide pool-specific CD4 T cell clones were tested for reactivity against autologous EBV-LCL pulsed with single peptides at 1 µM. After overnight coincubation, IFN-γ release was measured by ELISA. Of the 5 neopeptide pool-specific T cell clones from donor HD1 (upper panel), one clone reacted against peptide DNMT3A-FPV-H (blue symbols), one clone recognized peptide NUP98::NSD1-TTT (red symbols) and three clones were specific for peptide FLT3-GKV (yellow symbols). Clone 1.9.H7^FLT3/KIT^ recognized peptide KIT-ITK in addition to FLT3-GKV. All 8 neopeptide pool-specific T cell clones from donor HD3 (lower panel) reacted against the RUNX1-CAQ peptide (green symbols). The three T cell clones that reacted against the polymorphic peptide pool were specific for H-Y antigen RPS4Y-EKT as represented by clone 1.3.D11^RPS4Y^ (grey symbols). Symbols represent mean ± SD of duplicate wells. Representative data from one of two independent experiments is shown.

To elucidate which peptides were recognized, CD4 T cell clones were tested against autologous EBV-LCL pulsed with single peptides (**Figure 3**). Of the 5 neopeptide-specific clones from donor HD1, one clone (1.5.F1^DNMT3A^) recognized peptide DNMT3A-FPV-H encoded by *DNMT3A-R882H*, one clone (1.7.E7^NUP98::NSD1^) reacted against peptide NUP98::NSD1-TTT created by the *NUP98::NSD1* fusion and three clones (1.5.C8^FLT3^, 1.8.H4^FLT3^ and 1.9.H7^FLT3/KIT^) were specific for peptide FLT3-GKV encoded by *FLT3-D835Y*. In addition to FLT3-GKV, clone 1.9.H7^FLT3/KIT^ also recognized peptide KIT-ITK encoded by *KIT-D816V*, which shares 20 amino acids with FLT3-GKV. All 8 neopeptide-reactive T cell clones from donor HD3 (1.2.C2^RUNX1^, 1.2.D2^RUNX1^, 1.3.E8^RUNX1^, 1.4.B7^RUNX1^, 1.4.F6^RUNX1^, 1.4.G7^RUNX1^, 1.5.F10^RUNX1^ and 1.5.H7^RUNX1^) were specific for peptide RUNX1-CAQ derived from the long alternative reading frame created by frameshift mutations in *RUNX1*. TCR Vbeta analysis of RUNX1-reactive T cell clones by flow cytometry identified TRBV20-1 as Vbeta chain for clone 1.3.E8^RUNX1^, whereas the other 7 clones expressed TCR Vbeta chains that could not be identified by the antibodies in the TCR Vbeta Repertoire Kit. Lastly, all three T cell clones from donors HD2 (1.1.C3^RPS4Y^) and HD3 (1.3.D11^RPS4Y^ and 1.4.A3^RPS4Y^) recognizing the polymorphic peptide mix were specific for H-Y antigen LB-RPS4Y-1 (peptide RPS4Y-EKT) (represented by clone 1.3.D11^RPS4Y^ in **Figure 3**).

**Figure 4:**
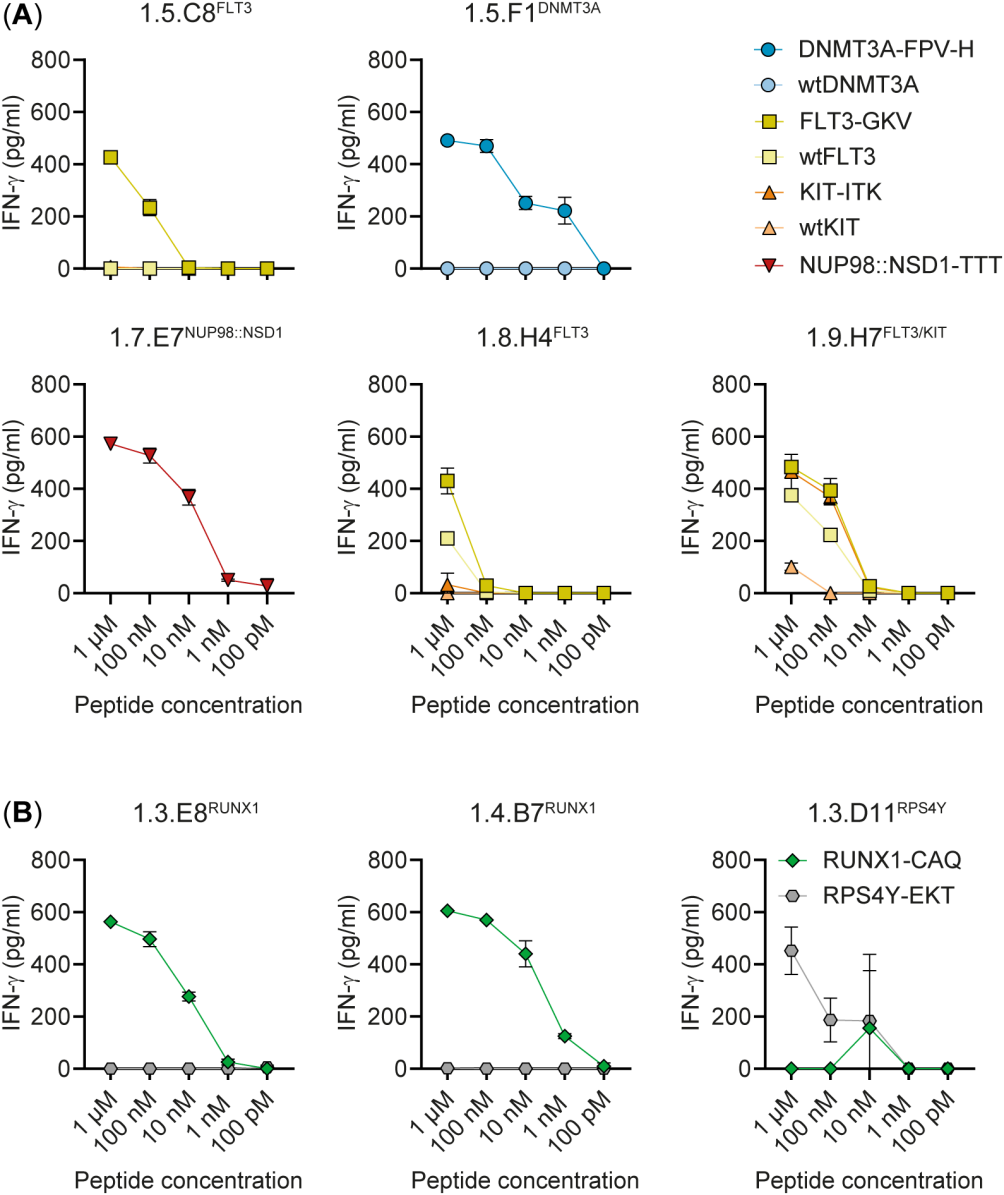
Affinity of CD4 T cell clones for titrated neopeptides. Neopeptide-specific T cell clones from donors HD1 **(A)** and HD3 **(B)** were tested against autologous EBV-LCL pulsed with titrated concentrations of neopeptides (dark symbols) or, if existing, wild-type peptides (wt, light symbols). After overnight coincubation, IFN-γ release was measured by ELISA. Specific recognition of neopeptides (DNMT3A-FPV-H, FLT3-GKV, KIT-ITK, NUP98::NSD1-TTT, RUNX1-CAQ) was observed for T cell clones 1.5.C8^FLT3^, 1.5.F1^DNMT3A^ and 1.7.E7^NUP98::NSD1^ and all RUNX1-specific clones represented by clones 1.3.E8^RUNX1^ and 1.4.B7^RUNX1^ (results for clones 1.2.C2^RUNX1^, 1.2.D2^RUNX1^, 1.4.F6^RUNX1^, 1.4.G7^RUNX1^, 1.5.F10^RUNX1^ and 1.5.H7^RUNX1^ are shown in **Supplementary Figure 2**). T cell clones 1.8.H4^FLT3^ and 1.9.H7^FLT3/KIT^ were also reactive against the wild-type peptide. Symbols represent mean ± SD of duplicate wells. Representative data from one of two independent experiments is shown.

Peptide specificity was further confirmed for all 13 neopeptide-specific T cell clones by recognition of EBV-LCL pulsed with titrated concentrations of mutated peptides and, if existing, wild-type peptides as controls. Clones 1.5.C8^FLT3^, 1.5.F1^DNMT3A^, 1.7.E7^NUP98::NSD1^, 1.8.H4^FLT3^ and 1.9.H7^FLT3/KIT^ all recognized mutated peptides (**Figure 4A**). No reactivity was observed against wild-type peptides, except for clones 1.8.H4^FLT3^ and 1.9.H7^FLT3/KIT^, which showed weak or similar reactivity against the wild-type FLT3 peptide. All RUNX1-specific T cell clones, represented by clones 1.3.E8^RUNX1^ and 1.4.B7^RUNX1^ (**Figure 4B**; other RUNX1-specific clones in **Supplementary Figure 2**), showed reactivity against the mutated RUNX1-CAQ peptide, but not against the RPS4Y-EKT negative control peptide, whereas clone 1.3.D11^RPS4Y^ was specific for RPS4Y-EKT, but not RUNX1-CAQ. In conclusion, the data showed that CD4 T cell clones 1.5.C8^FLT3^ and 1.5.F1^DNMT3A^ specifically reacted against HLA class II-binding neopeptides encoded by *FLT3-D835Y* and *DNMT3A-R882H* hotspot mutations, respectively, whereas T cell clone 1.7.E7^NUP98::NSD1^ reacted against an HLA class II-binding neopeptide encoded by the *NUP98::NSD1* fusion gene and CD4 T cell clones 1.2.C2^RUNX1^, 1.2.D2^RUNX1^, 1.3.E8^RUNX1^, 1.4.B7^RUNX1^, 1.4.F6^RUNX1^, 1.4.G7^RUNX1^, 1.5.F10^RUNX1^ and 1.5.H7^RUNX1^ against an HLA class II-binding neopeptide created by frameshift mutations in *RUNX1*.

### HLA class II restriction of neopeptide-specific T cell clones

To determine HLA class II restriction of T cell clones, reactivity was tested against 20 EBV-LCL each expressing at least three of the 23 HLA class II alleles to which the neopeptides were predicted to bind (**Supplementary Table 3**). Candidate HLA class II restriction alleles were deduced from T cell recognition patterns of EBV-LCL pulsed with neopeptides (**Supplementary Figure 3**). T cell clones 1.5.C8^FLT3^, 1.7.E7^NUP98::NSD1^ and 1.3.D11^RPS4Y^ all reacted against 5 peptide-pulsed EBV-LCL sharing HLA-DRB1*03:01. HLA-DRB1*15:01, -DQB1*06:02 and -DQB1*06:03 were deduced as candidate restriction alleles for clone 1.5.F1^DNMT3A^, since all EBV-LCL expressing these alleles were recognized. Recognition patterns were less clear for RUNX1-reactive T cell clones, since only 4 out of 6 peptide-loaded EBV-LCL that were recognized expressed HLA-DQB1*03:02.

**Figure 5:**
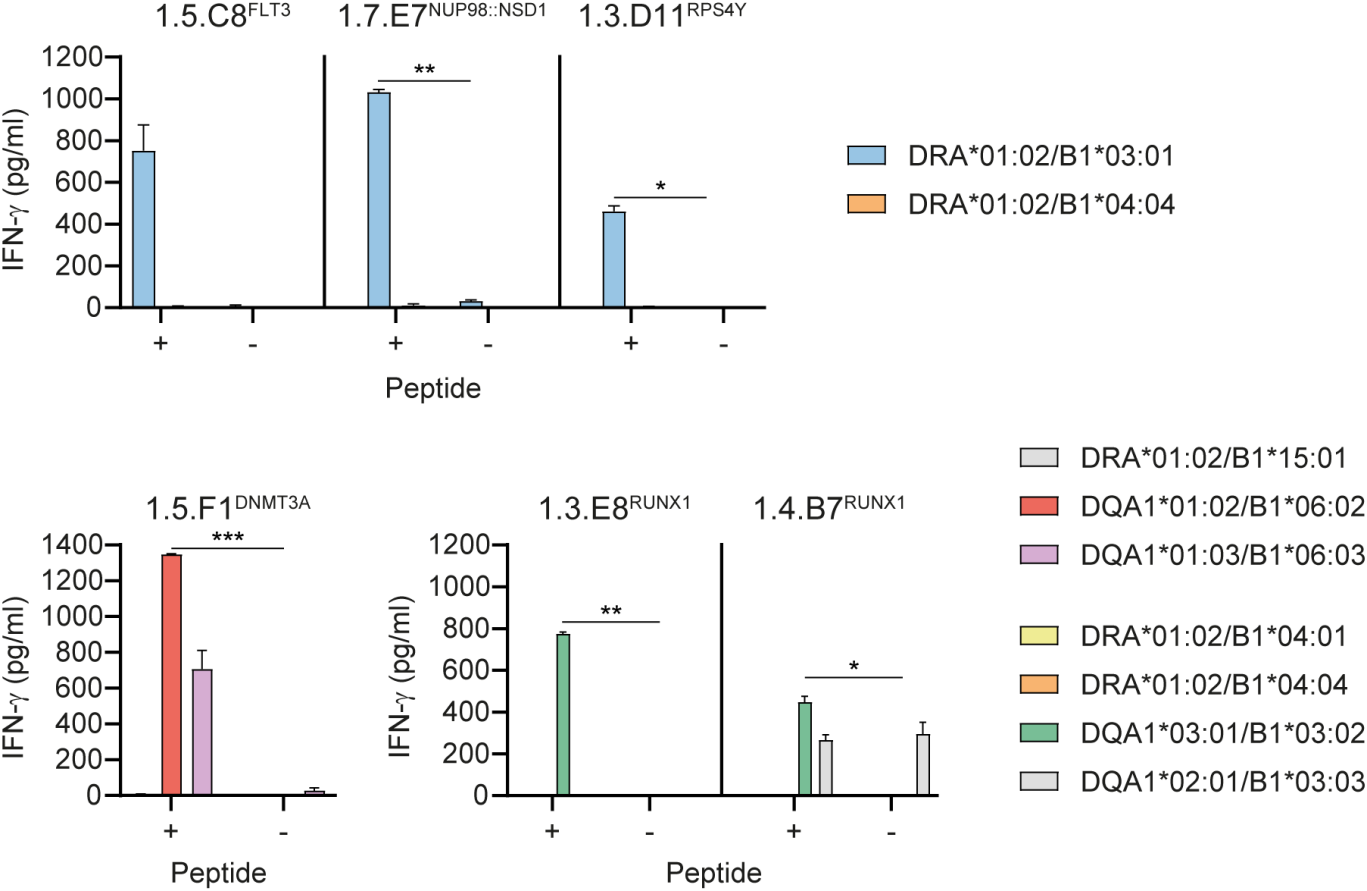
Validation of HLA class II restriction of neopeptide-specific CD4 T cell clones. Candidate HLA class II restriction alleles as deduced from T cell recognition patterns of peptide-loaded EBV-LCL were retrovirally introduced into K562. T cells were tested against K562 transduced with the indicated HLA-DRA, -DRB1, -DQA1 and -DQB1 alleles and pulsed with (+) or without (–) 1000 nM neopeptide. After overnight coincubation, IFN-γ release was measured by ELISA. HLA-DRA*01:02/B1*03:01 (blue bars) was validated as restriction allele for T cell clones 1.5.C8^FLT3^, 1.7.E7^NUP98::NSD1^ and 1.3.D11^RPS4Y^. T cell clone 1.5.F1^DNMT3A^ reacted against peptide-pulsed K562 transduced with HLA-DQA1*01:02/B1*06:02 (red bars) and to a lesser extent against peptide-pulsed K562 transduced with HLA-DQA1*01:03/B1*06:03 (purple bars), whereas K562 transduced with HLA-DRA*01:02/B1*15:01 (grey bars) was not recognized, thereby validating HLA-DQB1*06:02 and -DQB1*06:03 as restriction elements for clone 1.5.F1^DNMT3A^. HLA-DQA1*03:01/B1*03:02 (green bars) was confirmed as restriction element for T cell clones 1.3.E8^RUNX1^ and 1.4.B7^RUNX1^ (results for other RUNX1-specific T cell clones are shown in **Supplementary Figure 4**). T cell clone 1.4.B7^RUNX1^ also recognized K562 transduced with HLA-DQA1*02:01/B1*03:03 (grey bars) irrespective of peptide loading. A two-sided paired t-test was performed to determine if T cell reactivity against peptide-pulsed HLA-transduced K562 was significantly higher than against HLA-transduced K562 without peptide (p < 0.05 *, p < 0.01 **, p < 0.001 ***). Symbols represent mean ± SD of duplicate wells in a single experiment.

HLA class II restriction was validated by measuring T cell reactivity against peptide-pulsed K562 transduced with candidate HLA class II alleles (**Figure 5**). HLA-DRB1*03:01 was confirmed as restriction allele for clones 1.5.C8^FLT3^, 1.7.E7^NUP98::NSD1^ and 1.3.D11^RPS4Y^, since these clones reacted against K562 transduced with HLA-DRA*01:02/B1*03:01 when loaded with the respective peptides. HLA-DQB1*06:02 and -DQB1*06:03, but not HLA-DRB1*15:01, were confirmed as restriction elements for clone 1.5.F1^DNMT3A^ as demonstrated by reactivity against peptide-pulsed K562 transduced with the HLA-DQ alleles. Finally, HLA-DQB1*03:02 was validated as restriction allele for RUNX1-specific T cell clones, since all clones were reactive against peptide-pulsed K562 transduced with HLA-DQA1*03:01/B1*03:02 (1.3.E8^RUNX1^ and 1.4.B7^RUNX1^ in **Figure 5**; other RUNX1-specific clones in **Supplementary Figure 4**). With the exception of clone 1.3.E8^RUNX1^, RUNX1-reactive clones also reacted against K562 transduced with HLA-DQA1*02:01/B1*03:03 in the absence of RUNX1-CAQ peptide, indicating cross-reactivity against another peptide in HLA-DQB1*03:03. In summary, the results demonstrated that HLA-DRA*01:02/B1*03:01 is the HLA class II restriction allele for FLT3-D835Y-, NUP98::NSD1- and RPS4Y-specific T cell clones, whereas the DNMT3A-R882H-specific T cell clone was restricted to HLA-DQA1*01:02/B1*06:02 and HLA-DQA1*01:03/B1*06:03 and the RUNX1-specific T cell clones to HLA-DQA1*03:01/B1*03:02.

### T cell recognition of endogenously processed HLA class II neopeptides

**Figure 6:**
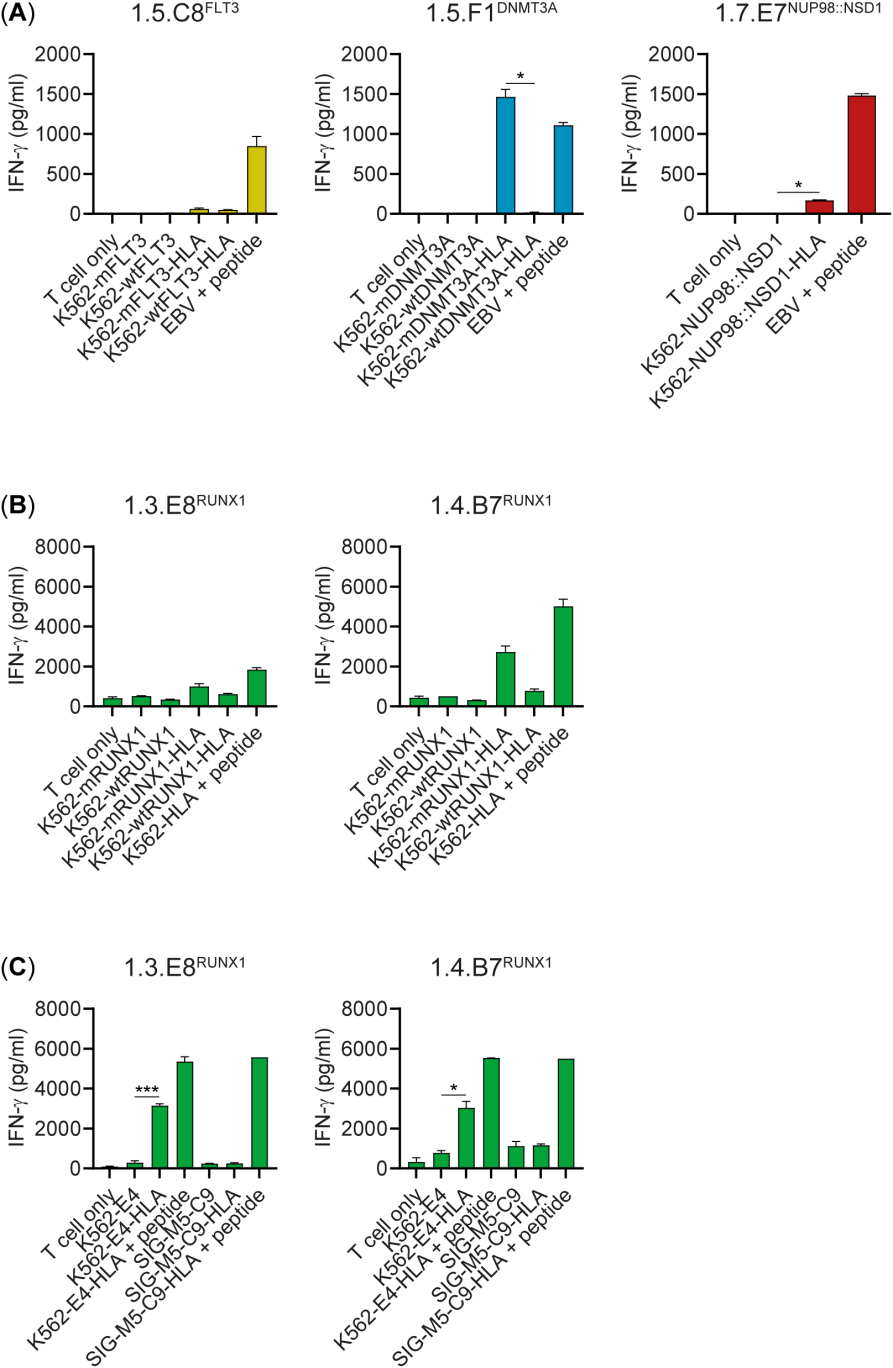
CD4 T cell recognition of endogenously processed neopeptides. K562 transduced with the relevant HLA class II restriction alleles as well as whole genes encoding mutated or wild-type proteins were coincubated overnight with neopeptide-specific CD4 T cell clones from donors HD1 **(A)** and HD3 **(B)**. Autologous EBV-LCL or HLA-transduced K562 (K562-HLA) were pulsed with 1 µM of neopeptide as positive controls. IFN-γ release was measured by ELISA. T cell clone 1.5.F1^DNMT3A^ recognized K562 transduced with HLA-DQA1*01:02/B1*06:02 and mutated *DNMT3A* (K562-mDNMT3A-HLA), but not K562 transduced with wild-type *DNMT3A* (K562-wtDNMT3A-HLA). T cell clone 1.5.C8^FLT3^ failed to react against K562 transduced with the relevant HLA class II allele and mutated gene (K562-mFLT3-HLA), while clone 1.7.E7^NUP98::NSD1^ secreted only low levels of IFN-γ. All RUNX1-specific T cell clones were more reactive against K562 transduced with HLA-DQA1*03:01/B1*03:02 and mutated *RUNX1* (K562-mRUNX1-HLA) than against K562 transduced with wild-type *RUNX1* (K562-wtRUNX1-HLA). Results are shown for clones 1.3.E8^RUNX1^ and 1.4.B7^RUNX1^ (results for the other RUNX1-specific clones are shown in **Supplementary Figure 5A**). A two-sided paired t-test was performed to determine if T cell reactivity against K562 transduced with HLA and the mutated gene was significantly higher than against K562 transduced with HLA and the wild-type gene (p < 0.05 *, p < 0.001 ***). Bars represent mean ± SD of duplicate wells in a single experiment. **(C)** RUNX1-specific T cell clones were tested against CML cell line K562 and AML cell line SIG-M5 in which *RUNX1* frameshift mutations were introduced by CRISPR-Cas9. The resulting genome-edited K562-E4 and SIG-M5-C9 cell lines were transduced with HLA-DQA1*03:01/B1*03:02 (K562-E4-HLA, SIG-M5-C9-HLA) and pulsed with 1 µM RUNX1-CAQ peptide as positive controls. After overnight coincubation, IFN-γ release was measured by ELISA. T cell clones 1.3.E8^RUNX1^, 1.4.B7^RUNX1^, 1.4.F6^RUNX1^ and 1.4.G7^RUNX1^ were more reactive against K562-E4-HLA than K562-E4 (clones 1.4.F6^RUNX1^ and 1.4.G7^RUNX1^ are shown in **Supplementary Figure 5B**). Clones 1.2.C2^RUNX1^, 1.2.D2^RUNX1^, 1.5.F10^RUNX1^ and 1.5.H7^RUNX1^ secreted only low levels of IFN-γ or did not react against K562-E4-HLA (**Supplementary Figure 5B**). None of the T cell clones recognized SIG-M5-C9-HLA. A two-sided paired t-test was performed to determine if T cell reactivity against K562-E4-HLA was significantly higher than against K562-E4, and reactivity against SIG-M5-C9-HLA significantly higher than against SIG-M5-C9 (p < 0.05 *, p < 0.001 ***). Bars represent mean ± SD of duplicate wells in a single experiment, except for peptide-pulsed SIG-M5-C9, which was tested in single wells.

To investigate whether the neopeptides can be endogenously processed and presented by HLA class II on the cell surface, we tested T cell reactivity against K562 transduced with the relevant HLA class II restriction alleles as well as whole genes encoding full-length mutated or wild-type proteins. Clone 1.5.F1^DNMT3A^ reacted against K562 transduced with HLA-DQA1*01:02/B1*06:02 and mutated *DNMT3A* (K562-mDNMT3A-HLA), but not wild-type *DNMT3A* (K562-wtDNMT3A-HLA) (**Figure 6A**). T cell clone 1.5.C8^FLT3^ failed to react against K562 transduced with mutated *FLT3* and the relevant HLA allele (K562-mFLT3-HLA), whereas clone 1.7.E7^NUP98::NSD1^ secreted only low levels of IFN-γ upon stimulation with K562 transduced with the *NUP98::NSD1* fusion gene and relevant HLA allele (K562-NUP98::NSD1-HLA). All 8 RUNX1-reactive T cell clones showed stronger reactivity against K562 transduced with HLA-DQA1*03:01/B1*03:02 and mutated *RUNX1* (K562-mRUNX1-HLA) than against K562 transduced with wild-type *RUNX1* (K562-wtRUNX1-HLA) (1.3.E8^RUNX1^ and 1.4.B7^RUNX1^ in **Figure 6B**; other RUNX1-specific clones in **Supplementary Figure 5A**). Four of the 8 T cell clones (1.3.E8^RUNX1^, 1.4.B7^RUNX1^, 1.4.F6^RUNX1^ and 1.4.G7^RUNX1^) also reacted against K562-E4 transduced with the relevant HLA-DQ allele (K562-E4-HLA) in which a homozygous *RUNX1* frameshift mutation was introduced by CRISPR-Cas9 (1.3.E8^RUNX1^ and 1.4.B7^RUNX1^ in **Figure 6C**; other RUNX1-specific clones in **Supplementary Figure 5B**). In contrast, AML cell line SIG-M5-C9 transduced with the relevant HLA-DQ allele (SIG-M5-C9-HLA) in which a similar heterozygous *RUNX1* frameshift mutation was introduced by CRISPR-Cas9 was not recognized. Altogether, our data showed that DNMT3A-FPV-H and RUNX1-CAQ are two neopeptides that can be endogenously processed and presented by HLA class II on the cell surface.

### Recognition of patient-derived AML cells by T cell clone 1.5.F1^DNMT3A^

**Figure 7:**
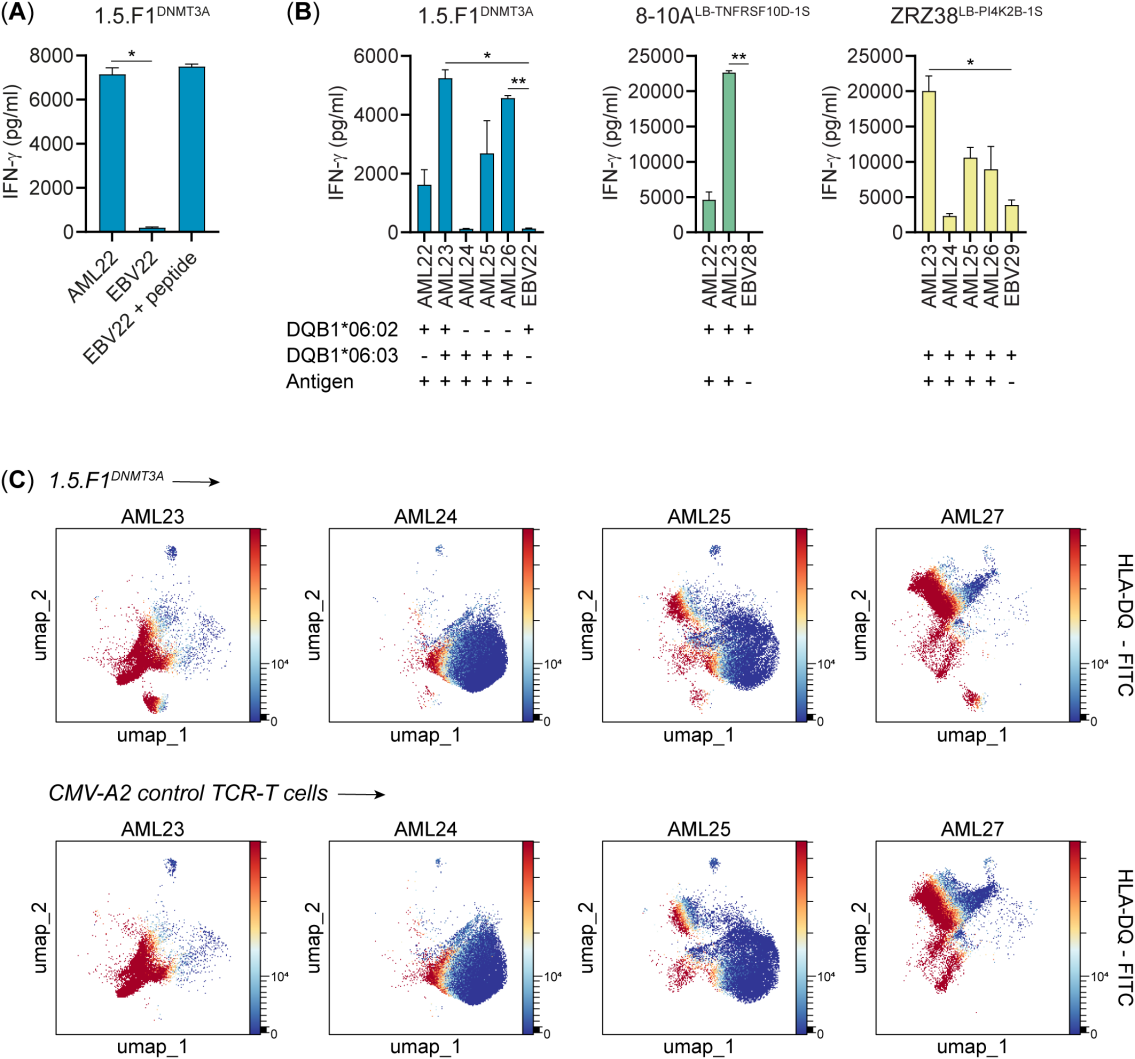
Targeting of patient-derived AML cells by T cell clone 1.5.F1^DNMT3A^. **(A)** T cell clone 1.5.F1^DNMT3A^ was tested for reactivity against HLA-DQB1*06:02 positive patient-derived AML cells with *DNMT3A-R882H*. To select AML cells with high HLA class II expression, cells were isolated by magnetic anti-PE beads after staining with a PE-conjugated antibody against HLA-DP prior to the test. EBV-LCL from the same patient were pulsed in the absence or presence of 1 µM of DNMT3A-FPV-H peptide and included as negative and positive control, respectively. After overnight coincubation, IFN-γ release was measured by ELISA. T cell clone 1.5.F1^DNMT3A^ showed specific recognition of AML cells, but not EBV-LCL. A two-sided paired t-test was performed to determine if T cell reactivity against AML cells was significantly higher than against EBV-LCL (p < 0.05 *, p < 0.01 **). Bars represent mean ± SD of duplicate wells in a single experiment. **(B)** T cell clone 1.5.F1^DNMT3A^ was tested for reactivity against multiple HLA-DQB1*06:02 or -DQB1*06:03 positive patient-derived AML samples with *DNMT3A-R882H* (n=5). HLA-DQB1*06:02-restricted T cell clone 8-10A and HLA-DQB1*06:03-restricted T cell clone ZRZ38 are reactive against minor histocompatibility antigens LB-TNFRSF10D-1S and LB-PI4K2B-1S, respectively, and were included as positive controls. For clone 1.5.F1^DNMT3A^, EBV22 was used as negative control. For clones 8-10A and ZRZ38, EBV-LCL positive for HLA-DQB1*06:02 and HLA-DQB1*06:03, respectively, but negative for the minor histocompatibility antigens were used as negative controls. After overnight coincubation, IFN-γ release was measured by ELISA. T cell clone 1.5.F1^DNMT3A^ reacted against 4 out of 5 AML, i.e. AML22, AML23, AML25 and AML26, but not against AML24. T cell recognition of AML24 was also low for positive control clone ZRZ38. A two-sided paired t-test was performed to determine if T cell reactivity against AML cells was significantly higher than against EBV-LCL (p < 0.05 *, p < 0.01 **). Bars represent mean ± SD of duplicate wells in a single experiment. **(C)** T cell clone 1.5.F1^DNMT3A^ was tested for lysis of HLA-DQB1*06:03 positive patient-derived AML samples with *DNMT3A-R882H* (AML23, AML24 and AML25) or wild-type *DNMT3A* (AML27) in a flow cytometry-based killing assay. AML samples were also tested for lysis by clone 1.5.F1^DNMT3A^ after pulsing with 1 µM DNMT3A-FPV-H peptide. All AML samples were also positive for HLA-A*02:01 and coincubated with CD8 T cells transduced with an HLA-A*02:01-restricted CMVpp65-specific TCR (CMV-A2 TCR-T cells) or mutated NPM1-specific TCR (mNPM1-A2 TCR-T cells) as negative and positive controls, respectively. After 48 hours of coincubation at a ratio of 1:1, samples were stained with PE-, APC- and FITC-conjugated antibodies against CD33, CD34 and HLA-DQ, respectively, viability dye Zombie Red to exclude dead cells and BV421-conjugated antibodies against CD3, CD8 and CD19 to exclude lymphocytes. Viable AML cells after coincubation with T cell clone 1.5.F1^DNMT3A^ (upper panels) or control CMV-A2 TCR-T cells (lower panels) were clustered by HLA-DQ expression. Each AML sample contained subpopulations with high HLA-DQ expression and subpopulations with no or low HLA-DQ expression. T cell clone 1.5.F1^DNMT3A^ did not kill AML cells with *DNMT3A-R882H* irrespective of HLA-DQ surface expression. Results were similar for negative control CMV-A2 TCR-T cells. Combined data from triplicate wells in a single experiment are visualized. Quantification of absolute numbers of viable AML cells is shown in **Supplementary Figure 6C**.

Since T cell clone 1.5.F1^DNMT3A^ showed reactivity against K562 transduced with mutated *DNMT3A* and the relevant HLA-DQ restriction allele, we tested reactivity of the clone against HLA-DQB1*06:02 or -DQB1*06:03 positive patient-derived AML samples with *DNMT3A-R882H*. We first tested reactivity against AML cells and, as negative control, EBV-LCL that were obtained from the same patient (**Figure 7A**). In this test, HLA class II-expressing AML cells were enriched by MACS, and EBV-LCL, generated from PBMCs when entering clinical remission after intensive chemotherapy, expressed high levels of HLA class II and wild-type *DNMT3A*. Clone 1.5.F1^DNMT3A^ specifically secreted IFN-γ upon stimulation with AML cells, but not EBV-LCL. Next, we tested clone 1.5.F1^DNMT3A^ against a larger panel (n=5) of HLA-DQB1*06:02 or -DQB1*06:03 positive patient-derived AML samples with *DNMT3A-R882H* (**Figure 7B**). In this experiment, AML cells were not enriched for HLA class II expression. Clone 1.5.F1^DNMT3A^ showed reactivity against all AML samples, albeit to different extents, and all AML samples were also recognized by positive control CD4 T cell clones for minor histocompatibility antigens (37). The only exception was AML24, which was not recognized by clone 1.5.F1^DNMT3A^ and weakly by the positive control T cell clone.

To determine whether T cell clone 1.5.F1^DNMT3A^ was able to kill AML cells with *DNMT3A-R882H*, clone 1.5.F1^DNMT3A^ was coincubated with patient-derived AML samples (n=4) for 48 hours, and numbers of viable HLA-DQ-expressing AML cells were measured in a flow cytometry-based killing assay (**Figure 7C** and **Supplementary Figure 6**). All 4 AML samples were subtyped as HLA-DQB1*06:03, and three AML carried *DNMT3A-R882H* (AML23, AML24, AML25), whereas one AML was negative for the mutation (AML27). In all AML samples, HLA-DQ expression was variable, with subpopulations expressing high levels and subpopulations expressing no or low HLA-DQ. The proportion of HLA-DQ-expressing cells was higher in AML23 and AML27 as compared to AML24 and AML25. The data showed that clone 1.5.F1^DNMT3A^ failed to induce specific lysis of AML cells with *DNMT3A-R882H* irrespective of HLA-DQ expression. Similarly, no lysis was observed after exogenous pulsing of AML cells with the DNMT3A-FPV-H peptide. In conclusion, we identified a DNMT3A-R882H neoantigen presented on patient-derived AML cells by HLA-DQB1*06:02 and -DQB1*06:03 that is recognized by a CD4 T cell clone, which is not able to kill AML cells.

## Discussion

In this study, we developed a strategy to isolate neoantigen-specific CD4 T cells after *in vitro* peptide stimulation, since T cell isolation by peptide-HLA class II tetramers is still challenging. Two neopeptides endogenously processed from *DNMT3A-R882H* and a long alternative reading frame created by *RUNX1* frameshift mutations were shown to be recognized by CD4 T cell clones. The DNMT3A-R882H peptide was also presented on patient-derived AML cells, thereby validating this peptide as public HLA class II neoantigen on AML.

*RUNX1* is mutated in 10-15% of patients with AML, of which one-third are frameshift mutations creating the same long alternative reading frame (38). We isolated 8 CD4 T cell clones specific for the RUNX1-CAQ peptide, which is located 30 amino acids from the C-terminus of the prolonged protein and shared by most *RUNX1* frameshift mutations. RUNX1-specific T cell clones reacted differently to K562-E4 and SIG-M5-C9, both expressing the same alternative reading frame. Of the 8 T cell clones, 4 clones recognized K562-E4, whereas none of the T cell clones reacted against SIG-M5-C9. Since the 8 T cell clones expressed at least two different TCR clonotypes, this lack of recognition by all T cell clones is unlikely to be caused by the TCRs, but probably attributable to intrinsic differences between the two cell lines. For instance, antigen expression may be lower on SIG-M5-C9 due to differences in HLA class II antigen processing and presentation, which is known to depend partly on the balance between HLA-DM and HLA-DO (39). Analysis of public gene expression data (40) demonstrated that the ratio of HLA-DO to HLA-DM in K562 and SIG-M5 is high, suggesting that both cell lines have the potential to present a broad repertoire of HLA class II peptides on their cell surface. Besides processing and presentation, lower antigen expression on SIG-M5-C9 may be caused by a heterozygous *RUNX1* mutation, whereas homozygous mutations are present in K562-E4. Moreover, T cell recognition could be influenced by differential expression of adhesion, costimulatory or inhibitory molecules on the cell surface of both cell lines. Although the exact reason remains unknown, T cell recognition of K562-E4 demonstrated that the RUNX1-CAQ peptide can be endogenously processed and presented on the cell surface. Whether the peptide is a neoantigen that can be targeted on AML by immunotherapy, for example by T cells engineered with TCRs of our RUNX1-specific clones, requires further testing of T cell clones against HLA-DQB1*03:02 positive patient-derived AML samples carrying *RUNX1* frameshift mutations, which were not available in our biobank and could not be easily collected.

*DNMT3A-R882* is a hotspot mutation in exon 23 occurring in approximately 15% of patients with AML. More than half of the mutations are *DNMT3A-R882H* mutations (32, 41). *DNMT3A* mutations are frequent in elderly people with clonal hematopoiesis of indeterminate potential (CHIP) or clonal cytopenia of undetermined significance (CCUS) who are at increased risk of developing hematological malignancies (42). This indicates that *DNMT3A* mutations are early driver mutations in preleukemic hematopoietic stem cells and that malignant transformation occurs upon accumulation of other cooperating mutations. *DNMT3A-R882H* is also often detectable in patients with AML in complete remission after chemotherapy (43–45), probably due to persistence of preleukemic stem cells after therapy. Our identified DNMT3A-R882H neoantigen may be used to target preleukemic stem cells by immunotherapy. Pre-emptive treatment with DNMT3A-R882H neoantigen-directed immunotherapy may be particularly attractive to prevent disease recurrence in patients with CHIP/CCUS who are in remission after treatment for AML.

Since all neoantigen-specific T cell clones in this study were isolated from healthy individuals, it remains unclear whether DNMT3A-R882H neoantigen-specific CD4 T cells are circulating in patients with AML. If present, neoantigen vaccination may be considered to induce or boost immune responses against *DNMT3A*-mutated AML. Personalized neoantigen vaccines have been shown to increase frequencies of neoantigen-specific T cells, and two recent studies reported clinical benefit of these vaccines (46, 47). Another approach, which is independent of circulating DNMT3A-R882H neoantigen-specific CD4 T cells, are bispecific T cell engagers. These engagers can be composed of a TCR or TCR-like domain that binds to a neoantigen-HLA complex and a domain that binds to CD3, thereby stimulating and redirecting CD8 as well CD4 T cell reactivity towards tumor cells (48). Alternatively, CD4 T cells can be genetically engineered with the DNMT3A-R882H neoantigen-specific TCR of clone 1.5.F1^DNMT3A^ and adoptively transferred to patients after *in vitro* expansion (49). Although these therapies may show clinical benefit, their efficacy can potentially be limited by the immunosuppressive tumor microenvironment in AML. AML cells can employ various mechanisms to suppress anti-tumor responses and have been shown to induce T cell dysfunction, upregulate expression of inhibitory molecules, secrete immunosuppressive soluble factors and downregulate HLA expression (50, 51). Therefore, the above immunotherapies may be more effective when combined with additional treatment targeting the immunosuppressive environment in AML.

Although cytotoxic CD4 T cells exist, T cell clone 1.5.F1^DNMT3A^ was able to release IFN-γ upon stimulation with patient-derived AML cells, but failed to induce lysis of these cells. Based on this lack of killing capacity, the TCR of clone 1.5.F1^DNMT3A^ is not expected to produce T cells with direct anti-tumor potential upon transfer to CD4 T cells. Our data also showed substantial intra- and inter-patient heterogeneity in HLA class II expression on AML cells, suggesting that AML cells with low or negative HLA class II expression may escape direct killing by CD4 T cells. Instead of mediating direct cytotoxicity, clone 1.5.F1^DNMT3A^ may promote anti-tumor immunity indirectly as T helper cell. CD4 T cells are known to contribute to anti-tumor responses by providing help to induction of cytotoxic CD8 T cells and stimulating their effector and memory functions (23, 24). Recent studies in mice convincingly demonstrated that CD4 T cells transduced with neoantigen-specific TCRs play an important role in eliminating major histocompatibility complex class II negative tumors when combined with CD8 T cells (52, 53). This provides a rationale to combine DNMT3A-R882H neoantigen-specific TCR-engineered CD4 T cells with neoantigen-specific CD8 T cells to treat AML. No HLA class I-restricted TCRs for DNMT3A neoantigens have been identified to date, but we previously isolated an HLA-A*02:01-restricted TCR for a NPM1 neoantigen, which upon transfer to CD8 T cells effectively targets AML *in vitro* and *in vivo* (15). Since *DNMT3A-R882* and *NPM1* mutations often co-occur (11), it is worth exploring whether combined transfer of CD8 T cells with the NPM1-specific TCR and CD4 T cells with the DNMT3A-specific TCR leads to more effective eradication of AML with *DNMT3A* and *NPM1* co-mutations as compared to NPM1-specific CD8 T cells alone.

In conclusion, we here identified an HLA class II neoantigen encoded by the well-known *DNMT3A-R882H* hotspot mutation that may become an important target for immunotherapeutic strategies to treat HLA-DQB1*06:02 or -DQB1*06:03 positive patients with AML.

## Data Availability

The raw data supporting the conclusions of this article will be made available by the authors, without undue reservation.
