# Supplementary material for: Mutated DNMT3A creates a public HLADQ- binding neoantigen on acute myeloid leukemia
Source: Front Immunol. 2025 Mar 13;16:1556121. doi: 10.3389/fimmu.2025.1556121 (PMC11947668; doi:10.3389/fimmu.2025.1556121)
Supplement: Supplementary file 1 [file DataSheet1.docx]

Supplementary Material

# Supplementary Tables

**Supplementary Table 1. Recurrent mutations in AML analyzed to encode neopeptides.**

| Gene | Mutation type | Position | Protein consequence |
| --- | --- | --- | --- |
| *DNMT3A* | Missense | *c.2645G>A*  *c.2644C>T* | p.Arg882His  p.Arg882Cys |
| *FLT3* | Missense | *c.2503G>T* | p.Asp835Tyr |
| *IDH1* | Missense | *c.395G>A*  *c.394C>T*  *c.394C>G*  *c.394C>A* | p.Arg132His  p.Arg132Cys  p.Arg132Gly  p.Arg132Ser |
| *IDH2* | Missense | *c.419G>A*  *c.515G>A* | p.Arg140Gln  p.Arg172Lys |
| *KIT* | Missense | *c.2447A>T* | p.Asp816Val |
| *NRAS* | Missense | *c.35G>A*  *c.38G>A* | p.Gly12Asp  p.Gly13Asp |
| *ASXL1* | 1 bp insertion | *c.1934dup* | p.Gly646Trpfs*12 |
| *CEBPA^1^* | 1 bp insertion  1 bp deletion | *c.68dup*  *c.247del* | p.His24Alafs*84  p.Gln83Serfs*77 |
| *NPM1* | 4 bp insertion | *c.860_863dup* | p.Trp288Cysfs*12 |
| *RUNX1^1^* | 1 bp insertion | *c.883dup* | p.Ser295Phefs*305 |

bp, base pair.

^1^Insertions or deletions at different positions within specific regions of *CEBPA* and *RUNX1* create different lengths of the same alternative reading frame. The indicated mutations create long reading frames between 76 and 83 amino acids for *CEBPA* and 304 amino acids for *RUNX1*.

**Supplementary Table 2. Recurrent gene fusions in AML analyzed to encode neopeptides^1^.**

| 5’ Gene | Exon | 3’ Gene | Exon |
| --- | --- | --- | --- |
| *CBFB* | *5* | *MYH11* | *34* |
| *KMT2A* | *9* | *MLLT3* | *2* |
| *KMT2A* | *9* | *MLLT3* | *6* |
| *KMT2A* | *10* | *MLLT3* | *6* |
| *NUP98* | *11* | *NSD1* | *7* |
| *NUP98* | *12* | *NSD1* | *7* |
| *PML* | *3* | *RARA* | *3* |
| *RUNX1* | *3* | *RUNX1T1* | *2* |
| *RUNX1* | *3* | *RUNX1T1* | *3* |

^1^The exon fusions of the two partner genes in known fusion transcripts were recurrently measured in 100 AML by whole transcriptome RNA-Seq (1).

**Supplementary Table 3. HLA class II typing of 20 EBV-LCL.**

| EBV-LCL | DRB1 | DRB3 | DRB4 | DRB5 | DQB1 | DPB1 |
| --- | --- | --- | --- | --- | --- | --- |
| HD3 | 03:01:01  04:04:01 | 01:01:02 | 01:03:01:01 |  | 02:01:01  03:02:01 | 04:01:01 |
| HD1 | 03:01  15:01 | 01:01 |  | 01:01:01 | 02:01:01  06:02:01 | 02:01  04:01/81:01 |
| 4 | 04:04  03:01 | 01:01 | 01:03 |  | 03:02  02:01 | 02:01  01:01 |
| 5 | 01:01:01  07:01:01 |  | 01:03:01:02 |  | 03:03:02  05:01:01 | 01:01/89:01  04:01/278:01/377:01 |
| 6 | 13:02  15:01 | 03:01 |  | 01:01 | 06:02  06:04 | 01:01  03:01 |
| 7 | 07:01:01  10:01:01 |  | 01:03:01:01 |  | 02:02:01  05:01:01 | 01:01:01  03:01/104:01 |
| 8 | 04:01  13:01 | 01:01 | 01:03 |  | 03:04  06:03 | 02:01  03:01 |
| 9 | 11:01  15:01 | 02:02 |  | 01:01 | 03:01  06:02 | 02:01:02  04:01 |
| 10 | 04:01:01  13:02:01 | 03:01:01 | 01:03:01:01 |  | 03:02/03:251  06:04/06:39 | 03:01/104:01/124:01  04:01/350:01 |
| 11 | 07  13:01/13:02/13:06 | 03 | 01/02/03 |  | 03:03  06:04/06:08/06:17 | 04:01  14:01 |
| 12 | 04:01/04:03/04:07  11:01/11:04/11:12 | 01/02/03 | 01/02/03 |  | 03:01/03:02/03:03  03:02/03:19/03:04 | 04:01:01 |
| 13 | 11:01  13:01 | 02:02  03:01 |  |  | 03:01  05:01 | 04:02  40:01 |
| 14 | 01:01:01  13:02:01 | 03:01:01 |  |  | 05:01:01  06:09 | 05:01:01  10:01 |
| 15 | 04  07 |  | 01/02/03 |  | 02:02  03:01 | 03:01  04:01 |
| 16 | 15:01  03:01 | 01:01:01 |  | 01:01:01 | 06:03  02:01 | 04:02  04:01 |
| 17 | 01:01/01:07/01:04  07:01/07:03/07:04 |  | 01/02/03 |  | 02:01/02:02/02:04  05:01/05:07 | 11:01:01  14:01 |
| 18 | 03:01/03:04/03:05  15 | 02 |  | 01 | 03:01  06:02/06:11/06:14 | 02:01:02  04:01 |
| 19 | 11:04:01  15:02:01 | 02:02:01 |  | 01:02 | 06:01:01  06:03:01 | 02:01:02 |
| 20 | 07:01:01  01:01/01:04/01:05 |  | 01:01:01:01  01/02/03 |  | 05:04  02:02 | 11:01:01  04:02 |
| 21 | 09  15 |  | 01/02/03 | 01 | 03:03  06:02/06:11/06:14 | 02:01:02  04:01 |

## Supplementary Figures


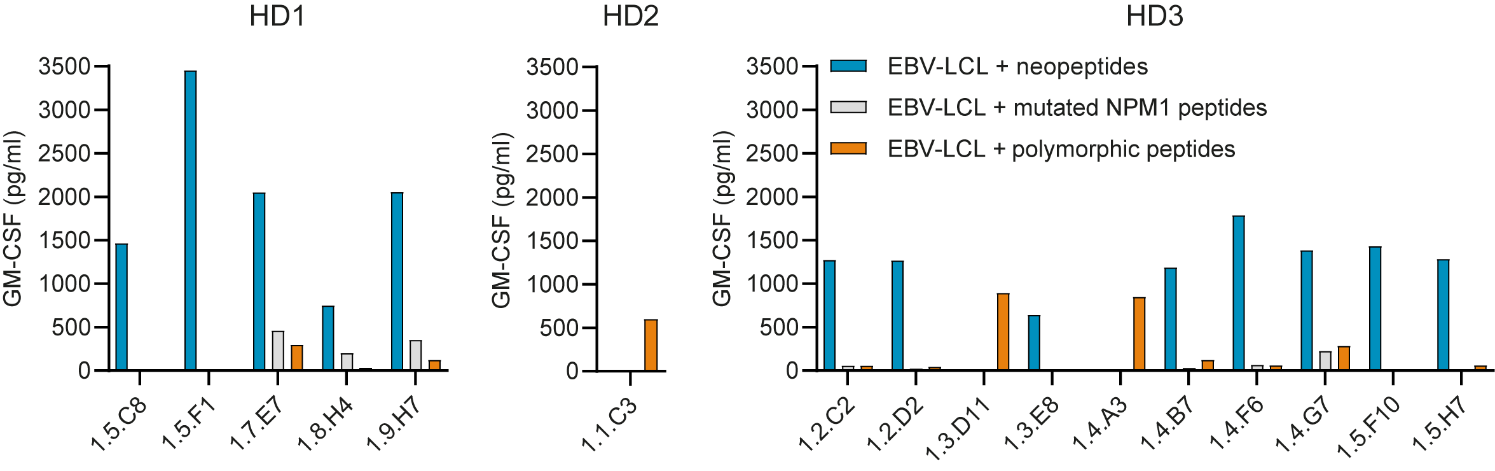


**Supplementary Figure 1. CD4 T cell clones recognizing neopeptide pools.** CD4 T cells from healthy female donors HD1-3 were stimulated with peptides *in vitro*, and activated CD4 T cells were sorted and single cell expanded as described in Figure 3. T cell clones were tested for reactivity against autologous EBV-LCL pulsed with three peptide mixes containing 13-16 neopeptides (blue bars), 8 mutated NPM1 peptides (grey bars) or 6-8 polymorphic peptides (orange bars) each at 100 nM. After overnight coincubation, GM-CSF release was measured by ELISA. Results are shown for 16 T cell clones that specifically reacted against peptide pools. The neopeptide pool was recognized by 5 T cell clones from donor HD1 and 8 clones from donor HD3. The polymorphic peptide pool was recognized by one T cell clone from donor HD2 and two clones from donor HD3. No T cell clones reacted against EBV-LCL loaded with mutated NPM1 peptides. Bars represent results from single wells in a single experiment.

**
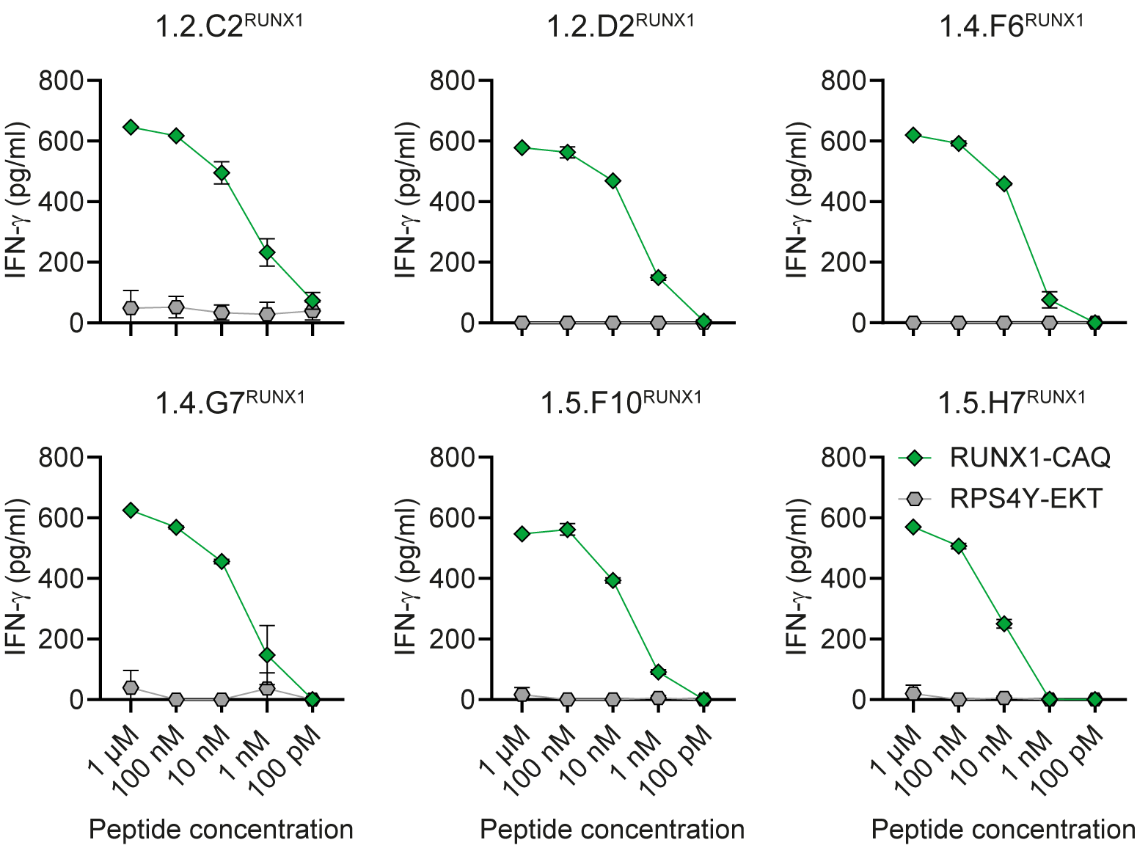
**

**Supplementary Figure 2.** **Affinity of CD4 T cell clones for titrated RUNX1-CAQ neopeptide.** T cell clones 1.2.C2^RUNX1^, 1.2.D2^RUNX1^, 1.4.F6^RUNX1^, 1.4.G7^RUNX1^, 1.5.F10^RUNX1^ and 1.5.H7^RUNX1^ from donor HD3 were tested against autologous EBV-LCL pulsed with titrated concentrations of RUNX1-CAQ neopeptide (green diamonds) as described in Figure 4. Specific recognition of RUNX1-CAQ was observed for all clones. Symbols represent mean ± SD of duplicate wells. Representative data from one of two independent experiments is shown.

**
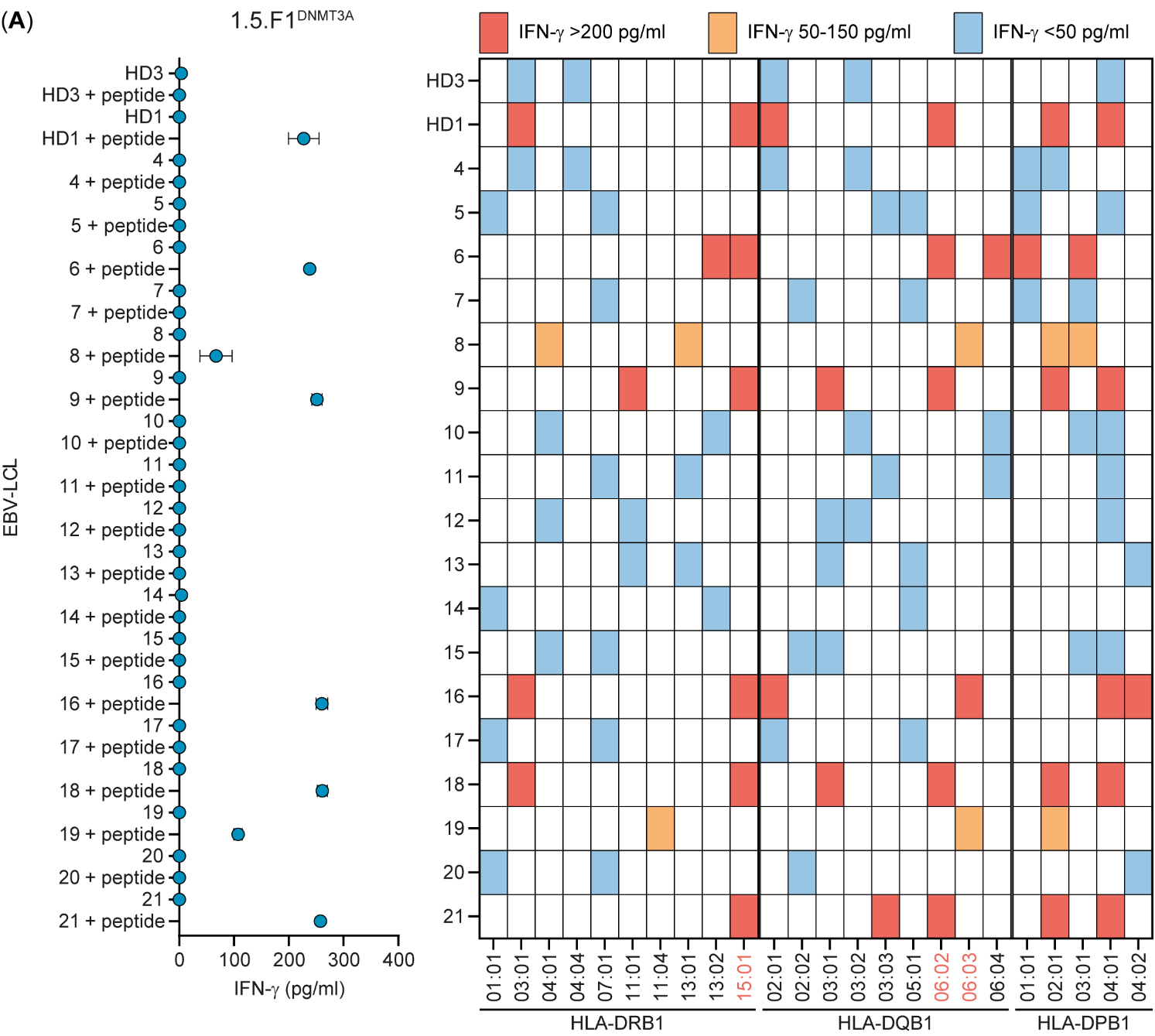
**

**Supplementary Figure 3. Determination of candidate HLA class II restriction alleles of neopeptide-specific CD4 T cell clones.** To determine HLA class II restriction, neopeptide-specific T cell clones were tested against a panel of 20 EBV-LCL pulsed in the absence or presence of 1 µM neopeptide. Positive EBV-LCL were included for each of the 23 common HLA-DRB1, -DQB1 and -DPB1 alleles as used for peptide selection as well as for HLA-DRB1*04:04, which is expressed by donor HD3. After overnight coincubation, IFN-γ release was measured by ELISA. Symbols represent mean ± SD of duplicate wells. Representative data from one of three independent experiments is shown. (**A**) T cell clone 1.5.F1^DNMT3A^ showed >200 pg/ml IFN-γ release upon stimulation with peptide-loaded EBV-LCL from donors HD1, 6, 9, 16, 18 and 21, whereas low levels of IFN-γ of 50-150 pg/ml were released upon stimulation with peptide-loaded EBV-LCL from donors 8 and 19 (left panel). The right panel depicts typing for the 24 HLA class II beta alleles in the panel of 20 EBV-LCL. Colored rectangles represent peptide-loaded EBV-LCL inducing IFN-γ release by clone 1.5.F1^DNMT3A^ of >200 pg/ml (red), 50-150 pg/ml (orange) or <50 pg/ml (blue). All 6 peptide-loaded EBV-LCL that were strongly recognized were positive for HLA-DRB1*15:01, whereas this allele was absent on EBV-LCL that were not recognized. HLA-DQB1*06:02 was shared between 5 out of 6 EBV-LCL and was absent on EBV-LCL that were not recognized. EBV-LCL from donors 8 and 19, which induced low levels of IFN-γ, were negative for HLA-DRB1*15:01 and -DQB1*06:02, but positive for HLA-DQB1*06:03. Based on these data, HLA-DRB1*15:01, -DQB1*06:02 and -DQB1*06:03 were candidate restriction alleles for clone 1.5.F1^DNMT3A^.


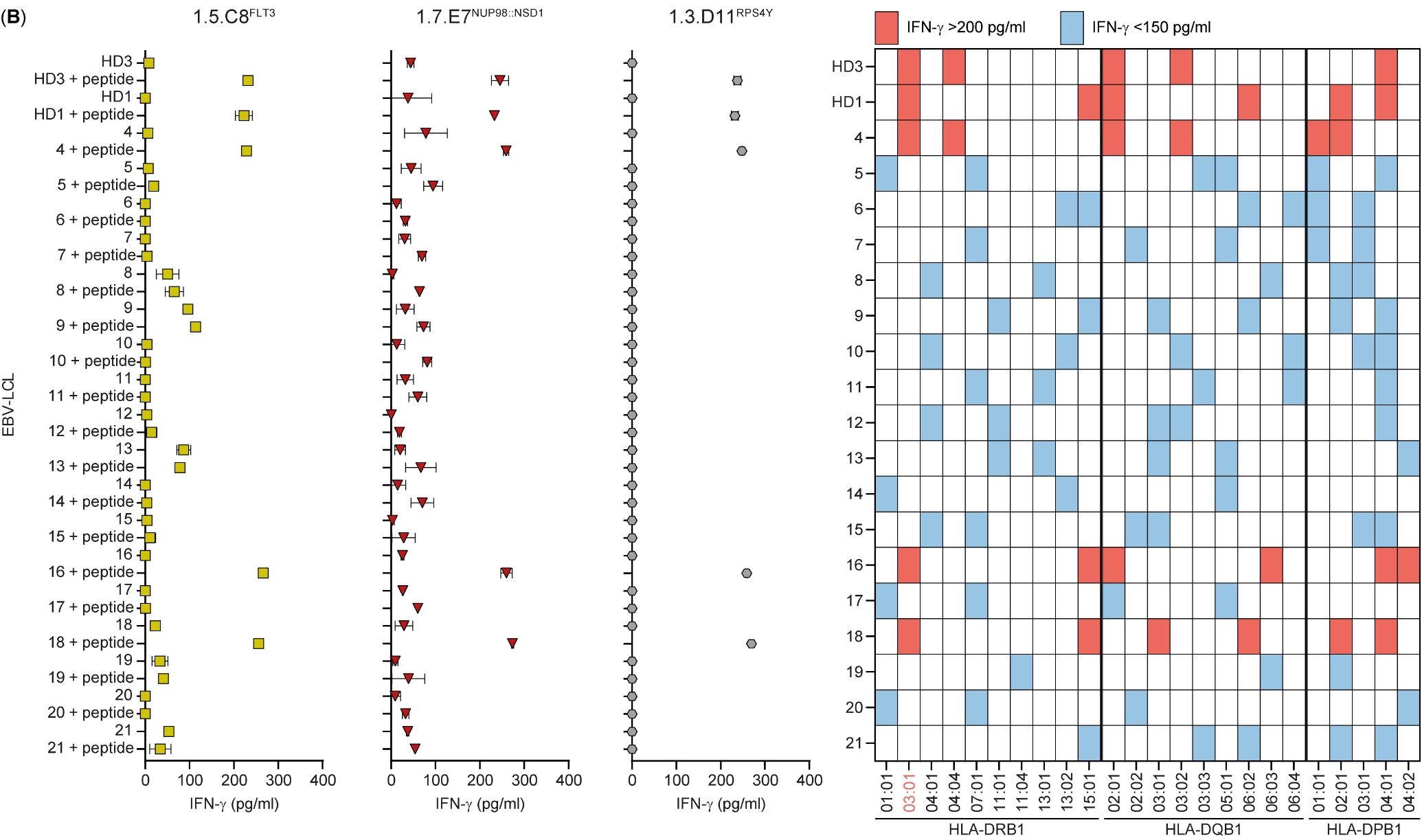


(**B**) T cell clones 1.5.C8^FLT3^, 1.7.E7^NUP98::NSD1^ and 1.3.D11^RPS4Y^ showed strong IFN-γ release (>200 pg/ml, red rectangles) upon stimulation with peptide-loaded EBV-LCL from donors HD3, HD1, 4, 16 and 18. These 5 EBV-LCL all expressed HLA-DRB1*03:01, whereas this allele was absent on EBV-LCL that were not recognized, suggesting that these T cell clones were HLA-DRB1*03:01-restricted.


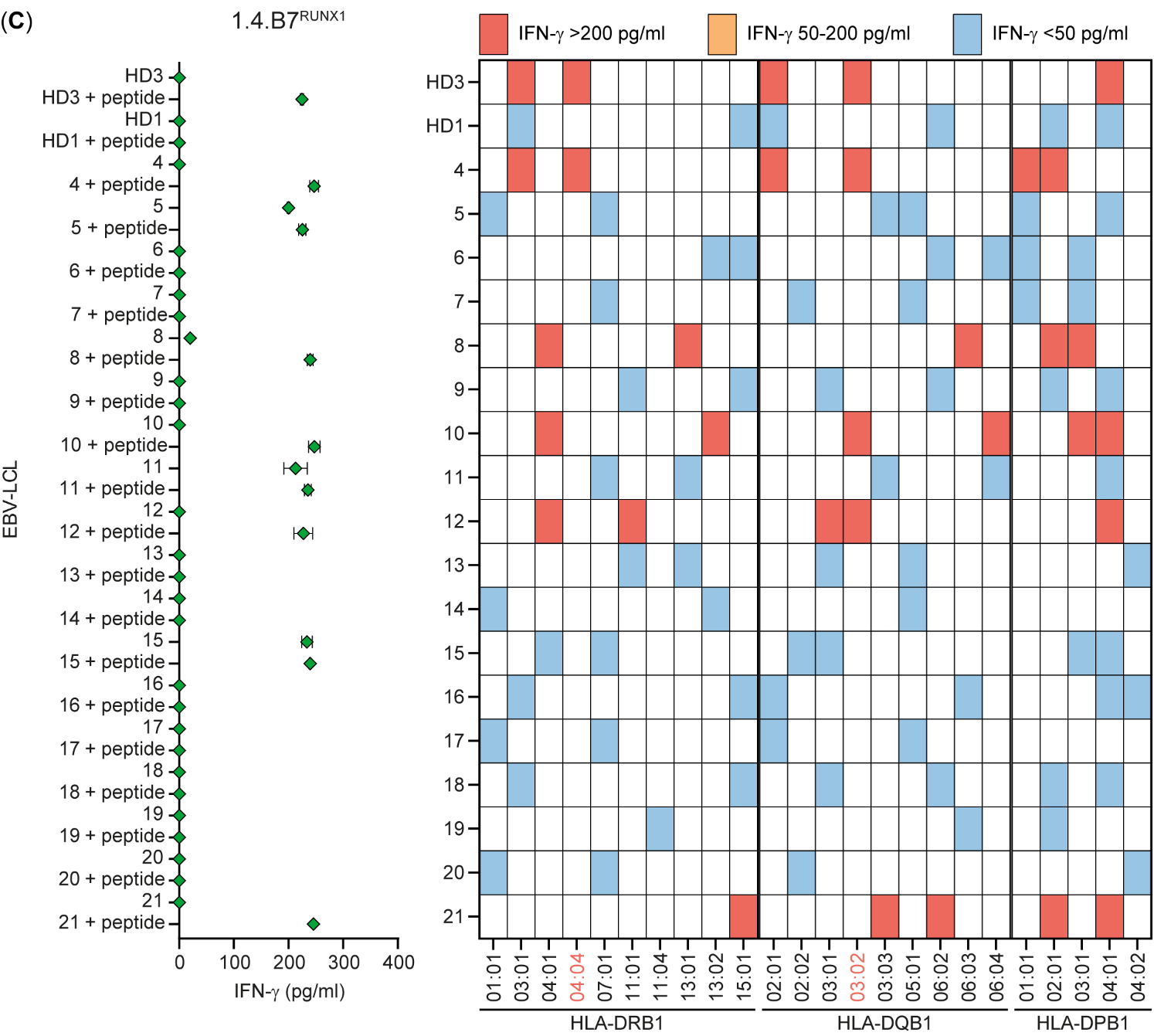


(**C**) All RUNX1-specific T cell clones, with the exception of clone 1.3.E8^RUNX1^, showed similar recognition patterns as represented by T cell clone 1.4.B7^RUNX1^. The T cell clones were strongly reactive against peptide-pulsed EBV-LCL from donors HD3, 4, 8, 10, 12 and 21. Of these 6 EBV-LCL, 4 shared HLA-DQB1*03:02 and two shared HLA-DRB1*04:04, while these alleles were absent on EBV-LCL that were not recognized. Based on these data, HLA-DQB1*03:02 was the candidate restriction element of RUNX1-specific T cell clones. All RUNX1-specific T cell clones, except for clone 1.3.E8^RUNX1^, reacted against EBV-LCL from donors 5, 11 and 15 irrespective of peptide pulsing.


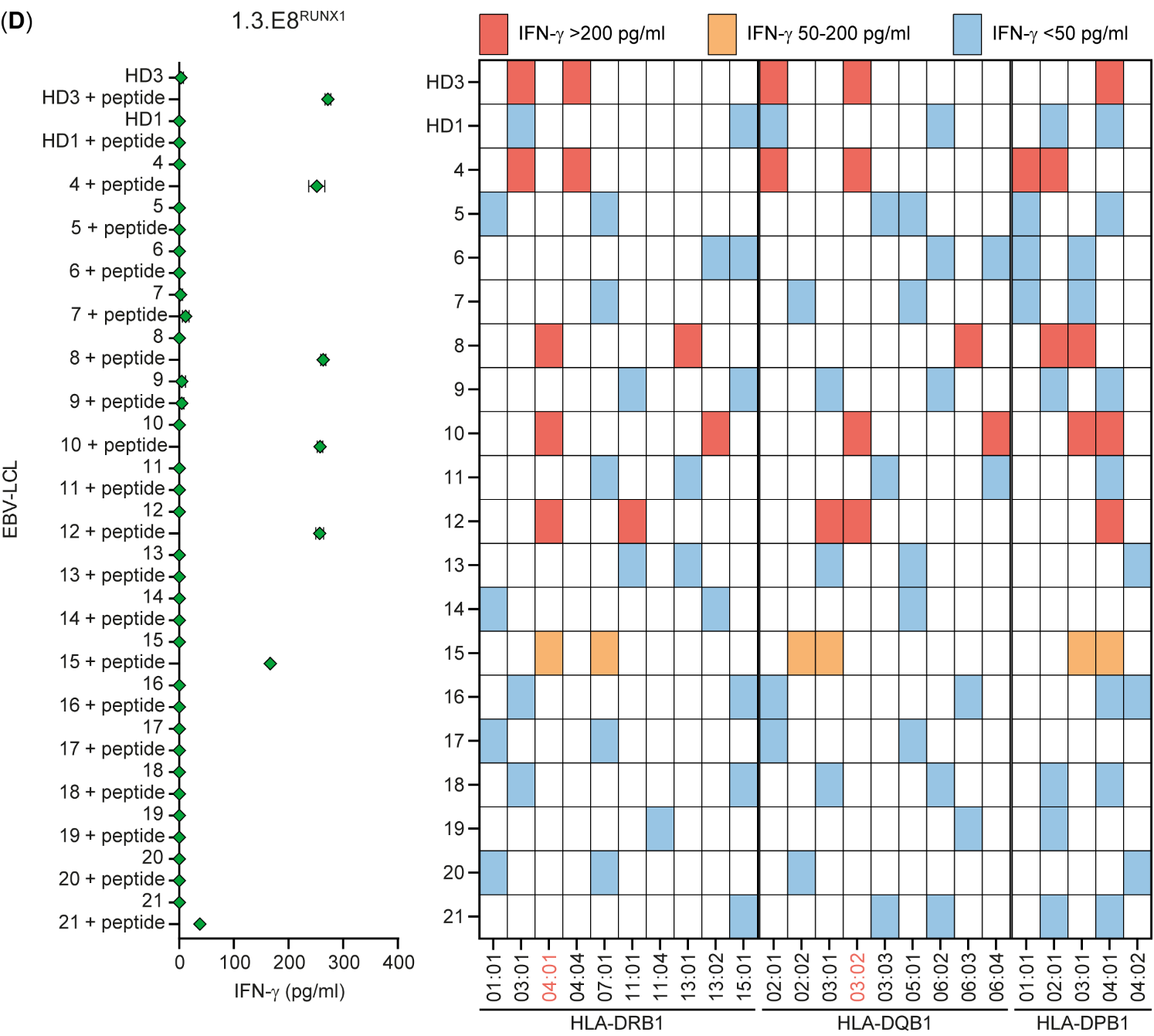


(**D**) T cell clone 1.3.E8^RUNX1^ showed a recognition pattern that was slightly different from other RUNX1-specific T cell clones, with strong recognition of peptide-loaded EBV-LCL from donors HD3, 4, 8, 10 and 12, whereas peptide-pulsed EBV-LCL from donor 15 was weakly recognized with IFN-γ release of 50-200 pg/ml. HLA-DQB1*03:02 and HLA-DRB1*04:01 were expressed by 4 and three of 5 strongly recognized EBV-LCL, respectively, suggesting that HLA-DQB1*03:02 was the candidate restriction allele for clone 1.3.E8^RUNX1^.

**
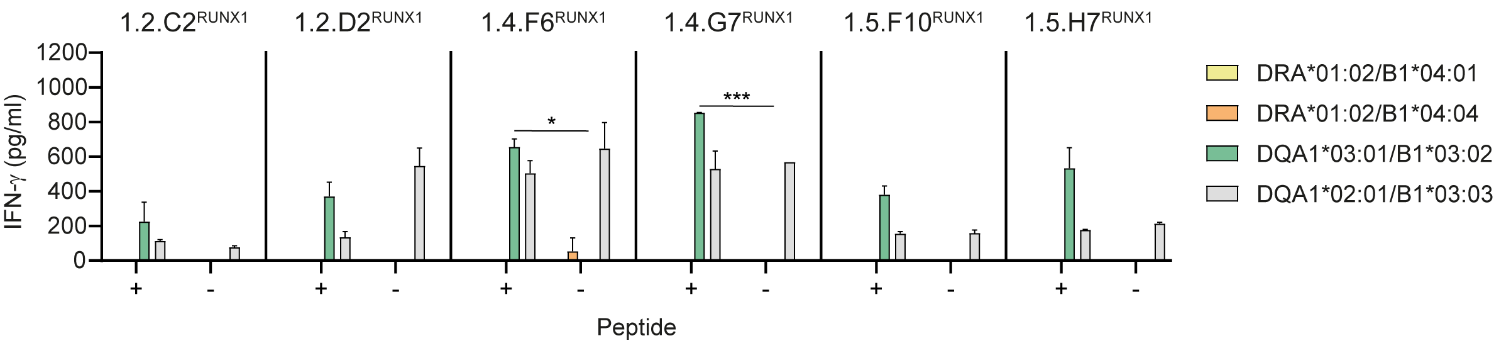
**

**Supplementary Figure 4. Validation of HLA class II restriction alleles for RUNX1-reactive T cell clones.** Based on T cell recognition patterns of peptide-loaded EBV-LCL as shown in Supplementary Figure 3, K562 cells were transduced with HLA-DRA*01:02/B1*04:01 (yellow bars), -DRA*01:02/B1*04:04 (orange bars), -DQA1*03:01/B1*03:02 (green bars) or -DQA1*02:01/B1*03:03 (grey bars) and used to stimulate RUNX1-specific T cell clones as described in Figure 5. HLA-DQA1*03:01/B1*03:02 was confirmed as restriction element for T cell clones 1.2.C2^RUNX1^, 1.2.D2^RUNX1^, 1.4.F6^RUNX1^, 1.4.G7^RUNX1^, 1.5.F10^RUNX1^ and 1.5.H7^RUNX1^. RUNX1-specific clones were cross-reactive against K562 transduced with HLA-DQA1*02:01/B1*03:03 irrespective of the RUNX1 neopeptide. A two-sided paired t-test was performed to determine if T cell reactivity against peptide-pulsed HLA-transduced K562 was significantly higher than against HLA-transduced K562 without peptide (p < 0.05 *, p < 0.01 **, p < 0.001 ***). Symbols represent mean ± SD of duplicate wells in a single experiment.

**
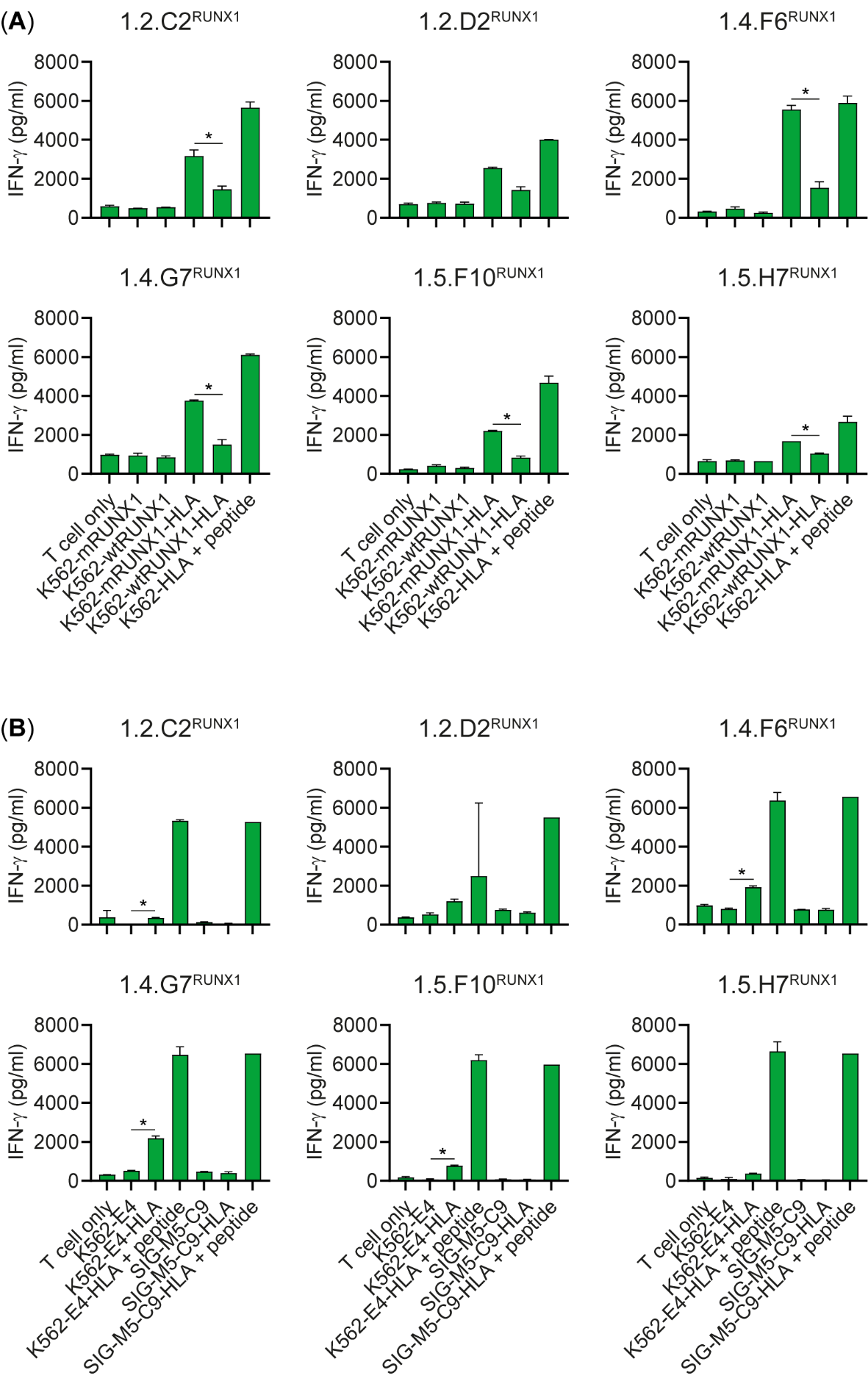
**

**Supplementary Figure 5. Recognition of cell lines with endogenous *RUNX1* mutations by CD4 T cell clones.** (**A**) T cell clones 1.2.C2^RUNX1^, 1.2.D2^RUNX1^, 1.4.F6^RUNX1^, 1.4.G7^RUNX1^, 1.5.F10^RUNX1^ and 1.5.H7^RUNX1^ were tested for recognition of K562 transduced with HLA-DQA1*03:01/B1*03:02 as well as whole genes encoding mutated or wild-type RUNX1 proteins as described in Figure 6A and B. All T cell clones were more reactive against K562 transduced with HLA-DQ and mutated *RUNX1* (K562-mRUNX1-HLA) than against K562 transduced with wild-type *RUNX1* (K562-wtRUNX1-HLA). A two-sided paired t-test was performed to determine if T cell reactivity against K562-mRUNX1-HLA was significantly higher than against K562-wtRUNX1-HLA (p < 0.05 *, p < 0.01 **, p < 0.001 ***). Bars represent mean ± SD of duplicate wells in a single experiment. (**B**) T cell clones 1.2.C2^RUNX1^, 1.2.D2^RUNX1^, 1.4.F6^RUNX1^, 1.4.G7^RUNX1^, 1.5.F10^RUNX1^ and 1.5.H7^RUNX1^ were tested against K562-E4 and SIG-M5-C9 cell lines in which *RUNX1* frameshift mutations were introduced by CRISPR-Cas9 as described in Figure 6C. T cell clones 1.4.F6^RUNX1^ and 1.4.G7^RUNX1^ were more reactive against K562-E4 transduced with HLA-DQA1*03:01/B1*03:02 (K562-E4-HLA) than K562-E4, whereas clones 1.2.C2^RUNX1^, 1.2.D2^RUNX1^, 1.5.F10^RUNX1^ and 1.5.H7^RUNX1^ showed low or no recognition of K562-E4-HLA. None of the RUNX1-specific T cell clones reacted against SIG-M5-C9 transduced with HLA-DQA1*03:01/B1*03:02 (SIG-M5-C9-HLA). A two-sided paired t-test was performed to determine if T cell reactivity against K562-E4-HLA was significantly higher than against K562-E4, and reactivity against SIG-M5-C9-HLA significantly higher than against SIG-M5-C9 (p < 0.05 *, p < 0.01 **, p < 0.001 ***). Bars represent mean ± SD of duplicate wells in a single experiment, except for peptide-pulsed SIG-M5-C9, which was tested in single wells.

**
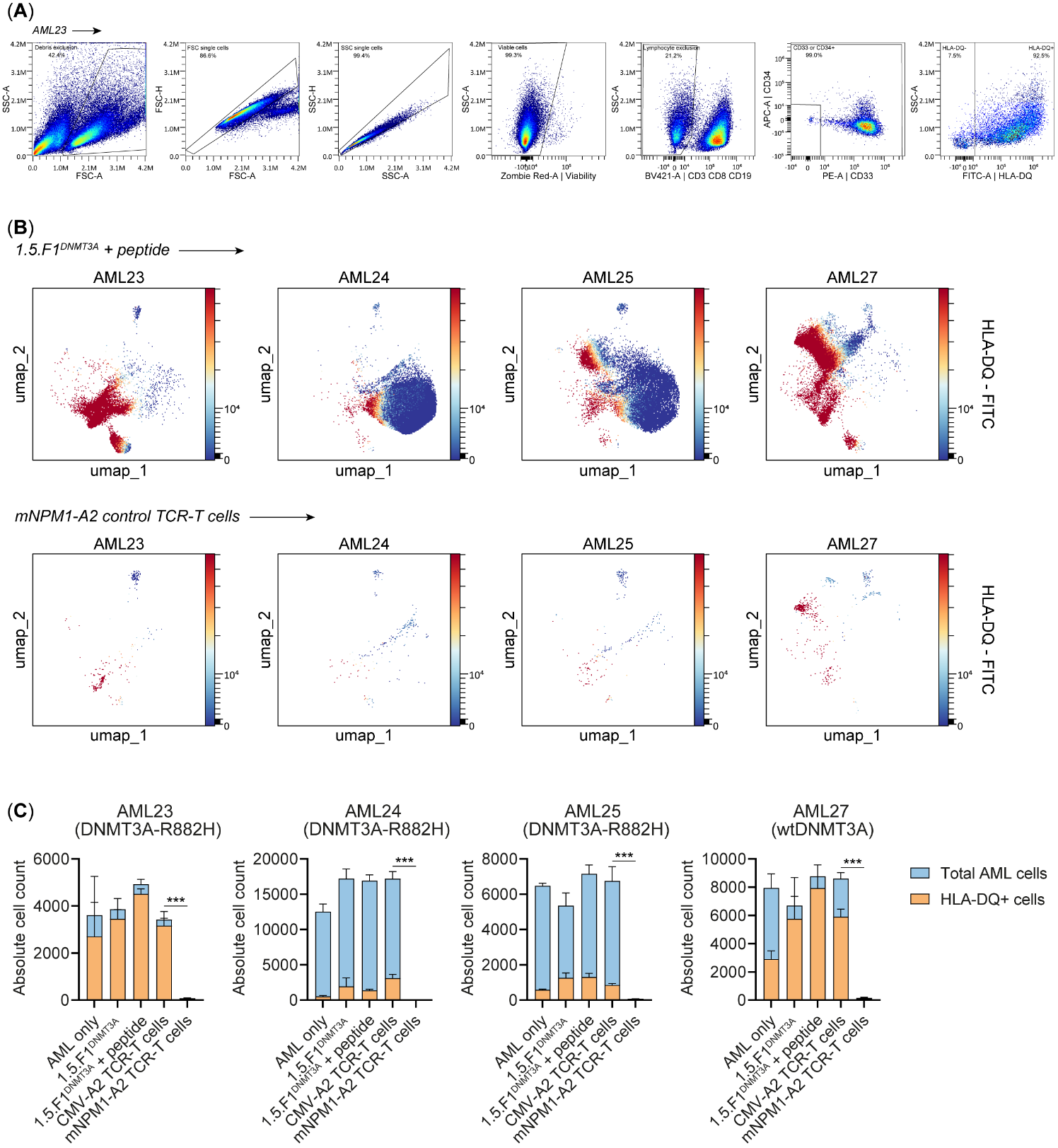
**

**Supplementary Figure 6. Flow cytometry-based killing assay of patient-derived AML cells.** T cell clone 1.5.F1^DNMT3A^ was tested for lysis of patient-derived AML cells (n=4) in a flow cytometry-based killing assay as described in Figure 7C. After 48 hours of coincubation, samples were stained with viability dye Zombie Red and antibodies against CD3, CD8, CD19, CD33, CD34 and HLA-DQ. (**A**) Depicted is the gating strategy for measuring HLA-DQ expression on viable AML cells for representative AML23. Debris and doublets were excluded using FSC and SSC, dead cells were excluded using Zombie Red viability dye and lymphocytes were excluded using antibodies against CD3, CD8 and CD19. AML cells were gated using antibodies against CD33 and CD34, and HLA-DQ expression was measured on viable AML cells. (**B**) Viable AML cells after coincubation with T cells were clustered by HLA-DQ expression as described in Figure 7C. Clustering of viable AML cells after pulsing with DNMT3A-FPV-H peptide and coincubation with T cell clone 1.5.F1^DNMT3A^ (upper panels) and clustering of viable AML cells after coincubation with positive control mNPM1-A2 TCR-T cells (lower panels) are shown. HLA-DQ expression was heterogenous within AML samples. (**C**) Absolute numbers of total viable AML cells (blue bars) and HLA-DQ positive viable AML cells (orange bars) pulsed in the absence or presence of DNMT3A-FPV-H peptide are shown after coincubation with T cell clone 1.5.F1^DNMT3A^. Clone 1.5.F1^DNMT3A^ did not mediate specific lysis of AML cells with *DNMT3A-R882H* irrespective of HLA-DQ expression and exogenous peptide pulsing. Results were similar for CMV-A2 TCR-T cells, whereas all AML cells were killed by mNPM1-A2 TCR-T cells. A two-sided unpaired t-test was performed to determine if lysis of absolute numbers of total AML cells was higher for clone 1.5.F1^DNMT3A^ (with and without peptide pulsing of target cells) and mNPM1-A2 TCR-T cells than for CMV-A2 TCR-T cells (p < 0.05 *, p < 0.01 **, p < 0.001 ***). Bars represent mean ± SD of triplicate wells in a single experiment.

**References**

1. Arindrarto W, Borràs DM, de Groen RAL, van den Berg RR, Locher IJ, van Diessen S, et al. Comprehensive diagnostics of acute myeloid leukemia by whole transcriptome RNA sequencing. Leukemia. 2021;35(1):47-61. (10.1038/s41375-020-0762-8).
